# An assessment of the physicochemical characteristics and essential oil composition of *Mentha longifolia* (L.) Huds. exposed to different salt stress conditions

**DOI:** 10.3389/fpls.2023.1165687

**Published:** 2023-04-18

**Authors:** Ruby Singh, Sajad Ahmed, Savita Luxmi, Garima Rai, Ajai Prakash Gupta, Rajendra Bhanwaria, Sumit G. Gandhi

**Affiliations:** ^1^ Council of Scientific & Industrial Research (CSIR)-Indian Institute of Integrative Medicine, Jammu, India; ^2^ Academy of Scientific and Innovative Research, Ghaziabad, India; ^3^ Department of Botanical and Environmental Sciences, Guru Nanak Dev University, Amritsar, India

**Keywords:** salt stress, growth, essential oil, physicochemical, biochemical, metabolites

## Abstract

Salt stress adversely influences growth, development, and productivity in plants, resulting in a limitation on agriculture production worldwide. Therefore, this study aimed to investigate the effect of four different salts, i.e., NaCl, KCl, MgSO_4_, and CaCl_2_, applied at various concentrations of 0, 12.5, 25, 50, and 100 mM on the physico-chemical properties and essential oil composition of *M. longifolia*. After 45 days of transplantation, the plants were irrigated at different salinities at 4-day intervals for 60 days. The resulting data revealed a significant reduction in plant height, number of branches, biomass, chlorophyll content, and relative water content with rising concentrations of NaCl, KCl, and CaCl_2_. However, MgSO_4_ poses fewer toxic effects than other salts. Proline concentration, electrolyte leakage, and DPPH inhibition (%) increase with increasing salt concentrations. At lower-level salt conditions, we had a higher essential oil yield, and GC–MS analysis reported 36 compounds in which (−)-carvone and D-limonene covered the most area by 22%–50% and 45%–74%, respectively. The expression analyzed by qRT-PCR of synthetic Limonene (LS) and Carvone (ISPD) synthetic genes has synergistic and antagonistic relationships in response to salt treatments. To conclude, it can be said that lower levels of salt enhanced the production of essential oil in *M. longifolia*, which may provide future benefits commercially and medicinally. In addition to this, salt stress also resulted in the emergence of novel compounds in essential oils, for which future strategies are needed to identify the importance of these compounds in *M. longifolia*.

## 1 Introduction

Under varying environmental conditions, plants are exposed to different stresses. There are many inbuilt mechanisms in plants by which they respond to these abiotic as well as biotic stresses, such as by altering the expression levels of several genes, cellular metabolism, growth rate, and morphology (Iqbal et al., 2021). In the framework of agronomy, these stress factors cause significant deviations in crop production. Among all stresses, salinity is known to be one of the most detrimental to crop growth and production (Kalefetoğlu and Ekmekci, 2005). In general, salinity is divided into primary and secondary salinities. A primary salinity is the result of natural processes such as weathering, rainfall, and strong winds that deposit salts over time on land and in water. Anthropogenic activities such as deforestation, land clearing, and excessive irrigation contribute to secondary salinity (Arif et al., 2020).

About one billion hectares of land surface area are altered by salinity, which approximates 7% of the entire land surface globally (Ghassemi et al., 1995; Qadir et al., 2014; Negacz et al., 2022). Although most of it is caused by natural geochemical progressions, it is estimated that 30% of irrigated land worldwide is contaminated by secondary salinization *via* anthropogenic activities (Machado and Serralheiro, 2017; Shahid et al., 2018; Hopmans et al., 2021). In a salinity-rich environment, higher ion concentration creates an osmotic imbalance, due to which water uptake in plants is negatively impacted (Khare et al., 2020; Ali et al., 2021; Ivy et al., 2022). It also reduces chlorophyll content, damages chloroplast ultrastructure, and reduces stomatal conductance, which hampers photosynthetic machinery, transpiration, and gas exchange (Arif et al., 2020; Thorne et al., 2020; El Sabagh et al., 2021).

Salinity stress changes the patterns of accumulation of secondary metabolites in plants (Gengmao et al., 2014; Yang and Guo, 2018). In *Rauvolfia tetraphylla* and *Catharanthus roseus*, the content of reserpine and vincristine alkaloids shows enhancement when subjected to salt stress, and it similarly enhances the essential oil (EO) content in *Salvia officinalis*, *Satureja hortensis*, and *Matricaria recutita* (Ashraf et al., 2018). Aromatic plants also accumulate secondary metabolites, including essential oil components, in response to salinity stress (Miransari et al., 2021).

As far as the soil salinity is concerned, sodium chloride (NaCl) and other salts like calcium chloride (CaCl_2_), magnesium chloride (MgCl_2_), sodium sulfate (Na_2_SO_4_), magnesium sulfate (MgSO_4_), sodium nitrate (Na_2_NO_3_), potassium nitrate (KNO_3_), etc. are also known to affect plant growth and development (Narjary et al., 2017). Soil salinity can be reversed, but it is time-consuming and relatively expensive. However, the plantation of salinity-resistant crops could be an effective and successful practice in such areas (Kulak et al., 2020).

*M. longifolia* (L.) Hudson is an important medicinal plant of the Lamiaceae (syn. Labiatae). It is a rapid-growing perennial aromatic herb with creeping rhizomes, commonly called ‘wild mint’ or ‘horse mint’ (Araghi et al., 2019; Moetamedipoor et al., 2021). It is widely distributed across Asia, Africa, Europe, Australia, and North America (Segev et al., 2012). *M. longifolia* is extensively used for its herbage and essential oils (Kumar et al., 2015; Jedrzejczyk and Rewers, 2018; Soilhi et al., 2019). Several pharmaceutical activities of both essential oils and extracts of *M. longifolia* have been investigated, including antimicrobial, antispasmodic, anticancer, antioxidant, hypertension, heart diseases, and most importantly, lowering the risk of cancer (Valko et al., 2006; Razavi et al., 2012; Hintz et al., 2015; Patonay et al., 2019). It also helps boost the immune system and fight secondary infections (Okut et al., 2017).

In the traditional systems of medicine all over the world, *M. longifolia* has been used for the treatment of gastrointestinal disorders, inflammatory disorders, respiratory diseases, infectious diseases, and menstrual disorders (Farzaei et al., 2017a). It is also used for the treatment of throat and mouth irritation (Farzaei et al., 2017b). In several food products like chewing gum, ice cream, candies, beverages, and non-vegetarian food items, the fresh leaves of *M. longifolia* are used as condiments (Krzyzanowska et al., 2011).

In saline areas, the selection of plants with salt tolerance is of great importance for bringing these areas into agricultural production. Higher economic value and desired chemical content are important targets in the selection of plants to be grown in these areas. However, studies regarding the physiological, biochemical, and molecular aspects of *M. longifolia* under different salt stress conditions are still lacking. A few investigations have been done on related phytochemical changes and secondary metabolites in *M. longifolia* grown under salinity conditions (El-Alakmy et al., 2017). The present study aimed to investigate the physiological, biochemical, molecular, and oil content of *M. longifolia* treated with NaCl, KCl, MgSO_4_, and CaCl_2_ at different concentrations (12.5, 25, 50, and 100 mM), whereas non-treated control plants were taken for comparison. Furthermore, the expression patterns of genes associated with the biosynthesis of mint essential oil were also analyzed at different salinity concentrations, and gene expression was correlated with the oil content.

## 2 Material and methods

### 2.1 Plant material

*M. longifolia* plant material was taken from the Chatha farm at CSIR-IIIM, Jammu. An herbarium specimen of *M. longifolia* was submitted to the Janaki Ammal Herbarium of the CSIR-Indian Institute of Integrative Medicine, Jammu, J&K, under voucher number 26772 after being morphologically identified by the plant taxonomist using taxonomical guidelines.

### 2.2 Pot preparation and treatments

**Figure 1:**
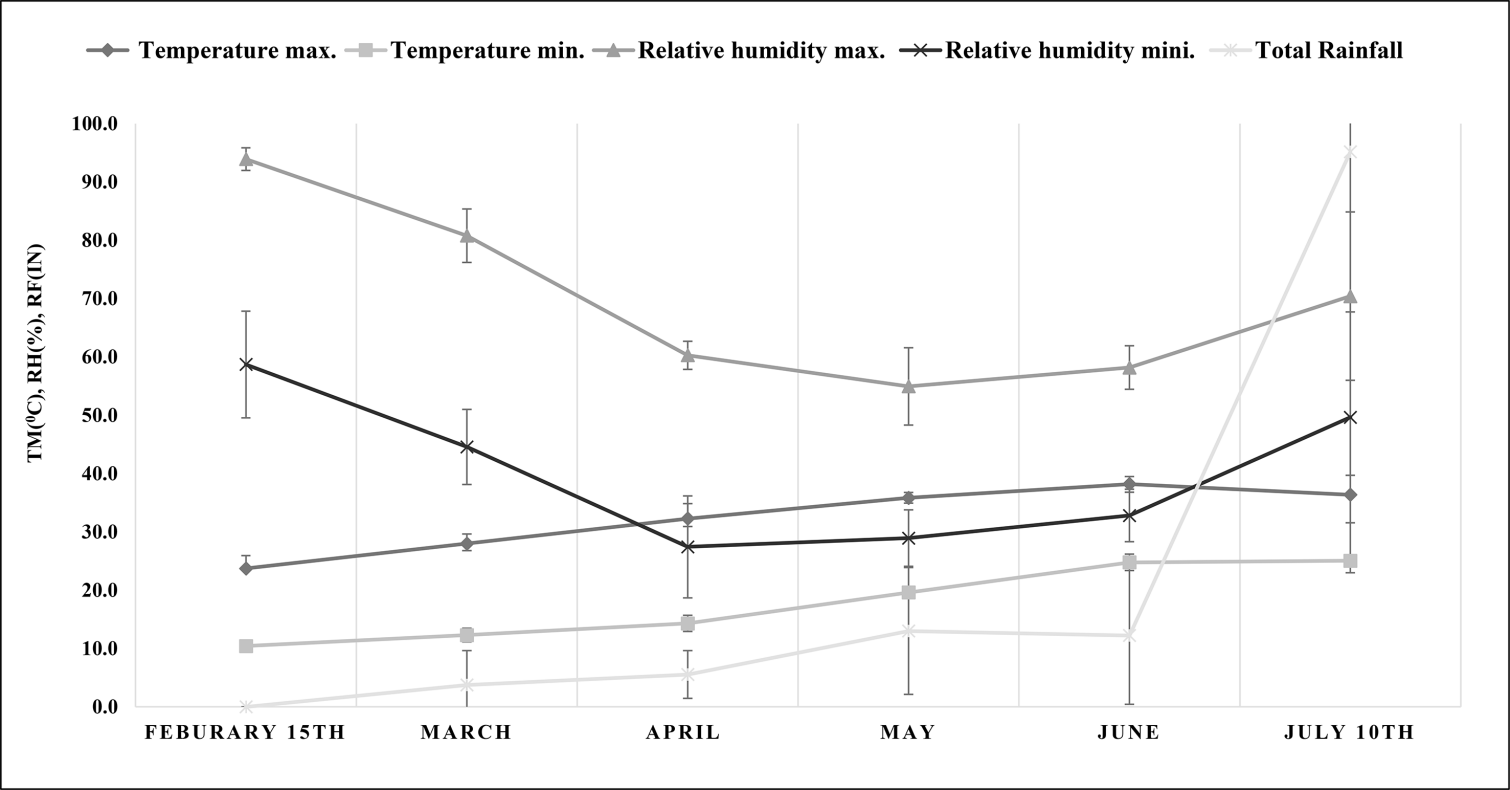
Meteorological data on maximum and minimum temperature (°C), maximum and minimum relative humidity (%) and total rainfall (in) during the experiment (from plant transplantation to harvesting). TM, temperature; RH, relative humidity; RF, rainfall.

The experiments were conducted in the greenhouse of the CSIR-Indian Institute of Integrative Medicine (CSIR-IIIM), located at an altitude of 327 m at 32.7315° N and 74.8505° E, from February to July 2021. The average monthly maximum and minimum temperature, relative humidity, and precipitation were recorded and are shown in **Figure 1** (data acquired from a weather station at the agronomy division of Sher-e-Kashmir University of Agricultural Sciences and Technology, Jammu). A 3:1 ratio of soil and manure was used to fill the pots (5 kg). Physical and chemical properties such as pH, electrical conductivity (EC), percentage of clay, sand, and silt, percentage of organic carbon, available nitrogen, available phosphorous, and available potassium (NPK) content were analyzed (**Table 1**) by methods described in our previous work (Singh et al., 2022). Water holding capacity (WHC) of soil was determined by the cup method as described by George et al. (2013). Four suckers of uniform maturity level and full-grown *M. longifolia* were planted in a plastic pot containing a soil mixture. Pots were kept in a greenhouse until a growing node appeared. Every treatment was given in three replicates (three different pots; one per replicate), and each pot had four plants. Plants were irrigated twice a week for two months after one month of transplantation to induce salt stress. In the experiment, four salts—NaCl, KCl, MgSO_4_, and CaCl_2_—were dissolved in water at four different concentrations: 12, 25, 50, and 100 mM, and an untreated control was also used.

**Table 1:** Experimental soil properties.

Sand (%)	53.33	Bulk density	1.38
Silt (%)	28	Organic carbon (%)	1.23
Clay (%)	18.66	Available nitrogen (kg/h)	263.424
pH	6.96	Available phosphorous(kg/h)	23.83
EC (dS m−1) WHC (%)	1.5 55	Available potassium (kg/h)	243.04

### 2.3 Morphological parameters

**Figure 2:**
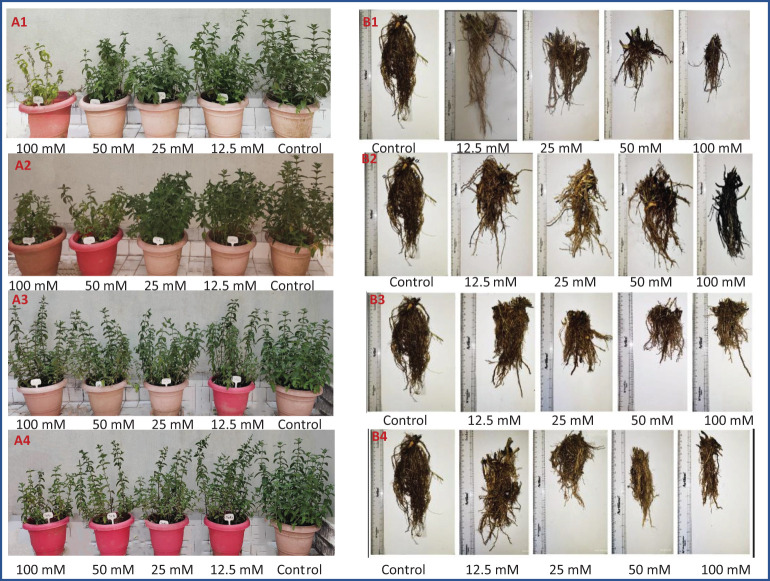
Morphology of *M. longifolia* plants (**A**—shoot and **B**—root) treated with salts. **(1)** NaCl, **(2)** KCl, **(3)** MgSO_4_, and **(4)** CaCl_2_.

After 15 d of salt treatment, when the plants were at the flowering stage, the plant height was measured and the number of branches of *M. longifolia* in each pot was counted (**Figure 2**). The plants were then harvested, and the roots and shoots of each treatment were separated. Both the root and shoot were washed twice with tap water, followed by distilled water. The fresh weight of the treated and control plants was taken using a weighing balance (Intwel, JW 24459). The plants were dried in an oven at 80°C for 48 h to achieve a constant dry weight before dry weight measurements.

### 2.4 Analyses of relative water content and electrolyte leakage

*M. longifolia* leaves of similar maturity levels were collected 24 h after salt treatment for relative water content (RWC) and electrolyte leakage (EL) analyses. The fresh weight of each leaf was taken before soaking the leaves in distilled water in sealed test tubes overnight at room temperature, after which the turgid weight was measured. The soaked leaves were dried in an oven for 24 h at 80°C when the weight was constant, and their dry weight was calculated. RWC was calculated using the formula given by Henson et al. (1981) and El-Bassiouny and Bekheta (2005).


$$\begin{array}{c} \text{RWC }=\;100\;\times \text{ (Fresh weight-Dry weight)}/\\ \text{(Turgid weight-Dry weight)} \end{array}$$

EL was determined by adopting the method of Dionisio-Sese and Tobita (1998). Briefly, 0.5 g of fresh leaves were chopped into small pieces (10 mm) and transferred to 20 ml of double-distilled water (ddH_2_O). The test tubes were incubated at 32°C in a water bath for 2 h. After the tubes had cooled down, the electrical conductivity (EC1) of the supernatant was determined using a conductivity meter (VSI-04ATC, VSI Electronics, India). After this, the samples were autoclaved for 20 min at 121°C, cooled to 25°C, and EC2 was determined. Then the electrolyte leakage was calculated using the following formula:


(EL)=EC1/EC2×100

### 2.5 Measurement of chlorophyll content

Photosynthetic pigments such as chlorophyll a (chl a), chlorophyll b (chl b), chlorophyll ab (chla + b), and carotenoids were measured by following the methods of Lichtenthaler (1987) and Voko et al. (2022). In 5 ml of ice-cold acetone, 0.10 g of fresh leaf tissue of similar maturity level was ground using a pestle and mortar. Then the extract obtained was filtered through filter paper (Whatman, GE Healthcare, UK), and an UV-visible spectrophotometer (Eppendorf, Biospectrophotometer, Eppendorf AG, 22331 Hamburg) was used to measure the absorbance at 470, 645, and 662 nm. The values obtained from the spectrophotometer were then calculated as described by Lichtenthaler (1987) for chl a, chl b, ch la + b, and carotenoids


Chlorophyll (a): 11.23(A at 662)-2.04(A at 645)

Chlorophyll (a): 20.13(A at 645)-4.19(A at 662)

$$\begin{array}{c} \text{Chlorophyll }\left(\text{a }+\text{ b}\right):\;7.05\left(\text{A at }662\right)\;\\ +\;18.09\left(\text{A at }645\right) \end{array}$$

$$\begin{array}{c} \text{Total carotenoids}:\;100\left(\text{A at }470\right)-1.09\text{chla}\\ -63.14\text{chlb}/124 \end{array}$$

### 2.6 Estimation of proline content

Fresh leaves of *M. longifolia* were taken for determination of proline content as described by Bates et al. (1973) with minor modifications. Briefly, 0.5 g of fresh leaves were homogenized with 10 ml of 3% sulfosalicylic acid. The homogenate obtained was filtered, then 1 ml of ninhydrin and 1 ml of glacial acetic acid were added, and the mixture was left undisturbed for an hour in a water bath set at 100°C. After incubation, it was cooled; the mixture was extracted with toluene, and the absorbance was measured at 520 nm. L-proline was used as a reference.

### 2.7 Estimation of DPPH inhibition percentage

Using the protocol described by Brand-Williams et al. (1995) and Xiao et al. (2020), the DPPH (1, 1-diphenyl-2-picryl-hydrazil) assay was carried out. The DPPH solution was prepared by dissolving 7.89 mg of DPPH in 100 ml of 99.50% ethanol and keeping it in the dark for 2 h. An ethanolic solution of ascorbic acid (1 mg/ml) was used as the standard. Approximately 200 µl of the extract was mixed with 1,000 µl of DPPH solution and 800 µl of the Tris–HCL buffer (pH 7.4), and the solution was kept for 30 min at room temperature (RT). The absorbance was then taken at 517 nm. Approximately 1,200 µl of ethanol and 800 µl of Tris–HCL buffer (pH 7.4) were mixed. Approximately 200 µl of this ethanol–Tris–HCL mix was used instead of a sample for measurement. The DPPH inhibition % was calculated with the following formula:


$$\begin{array}{c} \text{DPPH Inhibition percentage }=\text{ A }\left(\text{control}\right)\\ -\text{A }\left(\text{sample}\right)/\text{A }\left(\text{control}\right)\;\times \;100. \end{array}$$

### 2.8 Essential oil extraction

Hydro-distillation of fresh plant materials (100 g) was carried out for 3 h in a Clevenger (500 ml) along with water (200 ml). A pooled sample from each replicate was hydrodistilled. The essential oil was obtained after 3 h, dehydrated with anhydrous sodium sulfate salt, and stored in a refrigerator at 4°C.

### 2.9 Analysis of essential oil using gas chromatography and mass spectrometry (GC–MS)

Agilent 7890A gas chromatography linked to a 5875C mass spectrometer detector (XL MSD) with triple axis and MassHunter workstation software (USA) was used to analyze the samples. The column (Agilent) used was DB-5: 30 m × 0.25 mm i.d. × 0.25 μm film thickness. Helium operates as a carrier gas at a flow rate of 0.50 ml/min. The oven temperature of the gas chromatography was increased at a rate of 10°C/min from 200°C for 2 min to 280°C for 20 min. Approximately 1 μl of injection volume was done with a split ratio of 1:50. The mass spectra were captured in electron impact mode across the range of 50–600 amu at a scan rate of 0.5 s/scan and an ionization energy of 70 eV. The inlet and transfer line temperatures were maintained at 250°C. Wiley and NIST libraries were used for identifying components. Peak area percentages were electronically estimated without the use of correction factors from the TIC response.

### 2.10 Total RNA isolation, DNase treatment, and cDNA synthesis

TRIzol reagent (Ambion, Life Technologies, Carlsbad, USA) was used to isolate total RNA from *M. longifolia* leaves exposed to different salt concentrations following the manufacturer’s instructions. The integrity of isolated RNA was checked by 2% agarose gel electrophoresis. The NanoDrop Spectrophotometer (Thermo Fisher Scientific, Waltham, Massachusetts, USA) was used to analyze the absorbance ratio at 260/280 nm to determine the purity and concentration of total RNA. To remove any remaining genomic DNA contamination, the RNA samples were treated with DNase (Ambion TURBO DNA-free, Life Technologies, Carlsbad, CA, USA). A negative control PCR was carried out using the primers of the housekeeping gene to check for any potential DNA contamination in the DNase-treated RNA samples. cDNA was prepared from the DNase-treated RNA using a cDNA synthesis kit (Promega, Madison, WI, USA) in accordance with the manufacturer’s instructions. DNase-treated RNA samples were reverse-transcribed using a reverse transcriptase enzyme, oligo (dT) primers and 1 µg of DNase-treated RNA as a template.

### 2.11 qRT-PCR

Primer pairs (**Table 2**) were designed using the coding sequences (CDS) of the carvone and limonene biosynthesis genes of *M. longifolia*. The qRT-PCR experiments were carried out using the SYBR green master mix and the CFX96TM RT-PCR Detection System (Bio-Rad in Hercules, California, USA). The PCR reaction mixtures (10 μl) contained SYBR Green Master Mix 5.0 μl, Primer 1.0 µM (Saha Gene, Hyderabad, Telangana, India), appropriately diluted cDNA as a template, and the final volume of 10 μl was made up of MQ water. The thermoprofiles of the qRT-PCR reactions were included by preincubating at 95°C for 10 min, followed by 45 cycles of 3-step amplification (95°C for 10 s, 55°C for 10 s, and 72°C for 15 s) and melting. The reaction was normalized by using the primers for the 5.8 s gene as a reference. The fold change was then calculated using the 2^−ΔΔCT^ approach utilizing the threshold cycle (CT) (Ahmed et al., 2022).

**Table 2:** Table showing the details of primers used for gene expression analysis.

Name of primers		Sequences	Length	Melting temperature
5.8s (Reference gene)	Forward	CAACGGATATCTCGGCTCTC	20	55
	Reverse	TTGTGACACCCAGGCAGAC	19	
Geranyl pyrophosphate synthase (GPPS)	Forward	ATAAGCGGGCTGCATAG	17	55
	Reverse	CTGAATCTCCTCCTCGG	17	
Limonene synthase (LS)	Forward	GGTGGAGAAATACTGGGTT	19	55
	Reverse	GATCACCGTAATCAGAGCG	19	
Limonene-6-hydroxylase (L-6-H)	Forward	GGGATTTCATGGGAAACGA	19	55
	Reverse	ATCAGTCATTCCTTGAGGC	19	
Isopiperitenol dehydrogenase (ISPD)	Forward	GAGGCGCTATCATCTGC	17	55
	Reverse	GACACGCTGTTAACCCTAATC	21	

### 2.12 Statistical analysis

Each treatment was carried out in three replicates, and the data were represented as means. A one-way analysis of variance (ANOVA) was used to statistically evaluate the data using Excel 2019 and IBM SPSS Statistics version 26. The significant group was analyzed *via* Duncan’s multiple range analysis. Student t-tests were performed for a significant difference in the relative fold change of selected genes. Furthermore, the variances between the specific means were considered significant at p <0.05. PAST software and Origin Pro 2021 software were used to create the principal component analysis and correlation map, respectively.

## 3 Results

### 3.1 Morphology

The results showed that in *M. longifolia*, all four salts (NaCl, KCl, MgSO_4_, and CaCl_2_) led to significant changes in plant height (F = 3.17; p = 0.00), number of branches (F = 4.431; p = 0.00), number of inflorescences (F = 2.71; p = 0.01), plant fresh weight (F = 50.81; p = 0.00), and plant dry weight (F = 122.22; p = 0.00) (**Table 3**). In this case, F represents the variance of group mean values, and P represents the probability. All four (12.5, 25, 50, and 100 mM) concentrations of NaCl significantly reduced plant height by 16%, 12%, 21%, and 20%, respectively. With KCl treatments (12.5, 25, and 100 mM), the plant height was decreased by 7%, 2%, and 6%, whereas with 50 mM of KCl, a slight increase in height (2%) was observed when compared with the control. A 25 and 50 mM concentration of MgSO_4_ showed a 5% improvement in plant height. CaCl_2_ treatments (50 and 100 mM) resulted in a reduction in plant height of 6% and 13%, respectively. All salts significantly affected the number of branches. Maximum reduction (40%) occurred in 100 mM NaCl, followed by 19% in the CaCl_2_, and 14% in the KCl treatment. The number of branches improved (19%) with a 50 mM MgSO_4_ concentration. However, a 3% reduction in the number of branches was noticed at 100 mM MgSO_4_ concentration. As a result of salt stress, inflorescences significantly decreased. In comparison with the control group, 100 mM of KCl showed the highest reduction (52%), and 25 mM of MgSO_4_ showed the lowest reduction (8%) in the number of branches. Similarly, under salinity stress, the biomass (dry weight) of plants was also significantly reduced. Compared to the control, increasing NaCl concentrations reduced the total biomass of *M. longifolia* by 33%, 32%, 41%, and 58%, respectively. Similarly, plants treated with KCl showed reductions in total biomass of 47%, 53%, 59%, and 74% as compared to the control. In the case of CaCl_2_, too, a marked reduction in biomass occurred (59%, 57%, 45%, and 53%) with increasing salt concentrations. However, the effect of MgSO_4_ on total plant biomass was relatively subdued in comparison to NaCl, KCl, and CaCl_2_. Increased concentrations (12.5, 25, 50, and 100 mM) of MgSO_4_ reduced the biomass of *M. longifolia* by 34%, 15%, 50%, and 28%, respectively. In comparison with the control groups, all salts showed a deleterious effect on biomass, but a linear relationship between biomass and change in concentration was not observed. Here, the highest biomass was noted with a 25 mM MgSO_4_ treatment close to the control group, whereas the lowest biomass was noted with a 100 mM KCl treatment.

**Table 3:** Effects of different salinity levels on growth, development, and yield of *M. longifolia*.

Treatments	Plant height	No. of branches	No. of inflorescence	Fresh wt.	Dry wt.
Control	55.67 ± 5.51c–e	20.67 ± 0.58b	15.33 ± 0.58e	108.68 ± 7.75j	34.7 ± 2.63j
12.5 mM NaCl	46.34 ± 1.53ab	19.33 ± 2.89b	13 ± 1b–e	72.82 ± 2.01fg	22.92 ± 0.78g
25 mM NaCl	48.67 ± 2.52a–d	19 ± 1b	11.33 ± 0.58a–e	83.58 ± 8.31h	23.55 ± 0.36gh
50 mM NaCl	44 ± 20a	17 ± 2.65b	9 ± 1ab	74.63 ± 5.22fg	20.19 ± 1.12f
100 mM NaCl	44.34 ± 2.89a	12.33 ± 0.58a	7.67 ± 1.15a	56.73 ± 6.78bc	14.29 ± 1.51b
12.5 mM KCl	51.67 ± 9.71a–e	17.67 ± 3.06b	11 ± 2.65a–c	68.29 ± 1.68d–f	18.31 ± 1.11e
25 mM KCl	54.34 ± 6.11b–e	19.33 ± 2.08b	11.67 ± 1.15a–e	60.73 ± 1.75cd	16.03 ± 1.17c–d
50 mM KCl	56.67 ± 1.53c–e	18.67 ± 1.53b	9.33 ± 1.53a–c	50.2 ± 2.69b	14.18 ± 0.15b
100 mM KCl	52 ± 3a–e	17.67 ± 2.52b	7.33 ± 2.52a	42.32 ± 3.40a	8.73 ± 0.33a
12.5 mM MgSO4	51.34 ± 10.69a–e	18.67 ± 2.31b	10.67 ± 6.66a–d	80.62 ± 3.85gh	22.66 ± 0.84g
25 mM MgSO4	58.67 ± 1.53e	20.67 ± 0.58b	14 ± 1d–e	108.6 ± 1.32h	29.19 ± 0.12i
50 mM MgSo4	58.67 ± 1.53e	24.67 ± 2.52c	13.67 ± 0.58c–e	72.22 ± 2.90ef	17.13 ± 0.17de
100 mM MgSo4	55.34 ± 4.04c–e	20 ± 1b	13.67 ± 1.15c–e	96.31 ± 4.68i	24.98 ± 0.28h
12.5 mM CaCl_2_	53.67 ± 2.52b–e	20.67 ± 0.58b	12.67 ± 1.53b–e	70.5 ± 0.34ef	13.98 ± 0.18b
25 mM CaCl_2_	57.34 ± 2.89d–e	19 ± 2b	11.33 ± 3.21a–e	56.54 ± 3.31bc	14.84 ± 0.26bc
50 mM CaCl_2_	52 ± 2.65a–e	20 ± 1.73b	11.33 ± 1.53a–e	70.32 ± 6.16ef	18.79 ± 1.41ef
100 mM CaCl_2_	48.34 ± 0.58a–b	16.67 ± 3.51b	12.67 ± 3.21b–e	63.95 ± 4.4c–e	16.26 ± 0.15cd

Means in the same column by the same letter are not significantly different to the test of Duncan (α = 0.05).

### 3.2 Relative water content of leaves and electrolyte leakage

**Figure 3:**
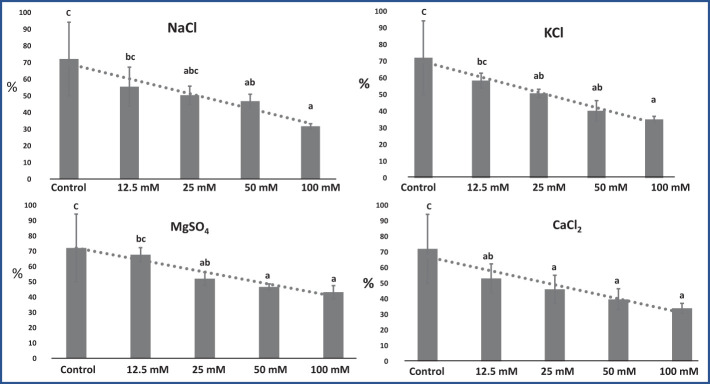
Effect of 12.5, 25, 50, and 100 mM concentrations of NaCl, KCl, MgSO_4_, and CaCl_2_ salts on leaf relative water content (%) in an *M. longifolia* leaf sample. The data are represented as mean ± standard deviation (n = 3). The different letters on the bar graph show the significantly different groups according to the Duncan test (α = 0.05).

RWC of leaves was significantly affected by all four salt conditions: NaCl (F = 4.73; p = 0.02), KCl (F = 5.83; p = 0.01), MgSO_4_ (F = 4.51; p = 0.02), and CaCl_2_ (F = 4.58; p = 0.02). RWC significantly decreased with increasing salt concentrations (**Figure 3**). RWC was found to be highest in the control group (72.19%) and lowest in the 100 mM NaCl group (31.81%). NaCl, KCl, and CaCl_2_ affected RWC to a greater extent as compared with MgSO_4_, which was found to be less harmful.

**Figure 4:**
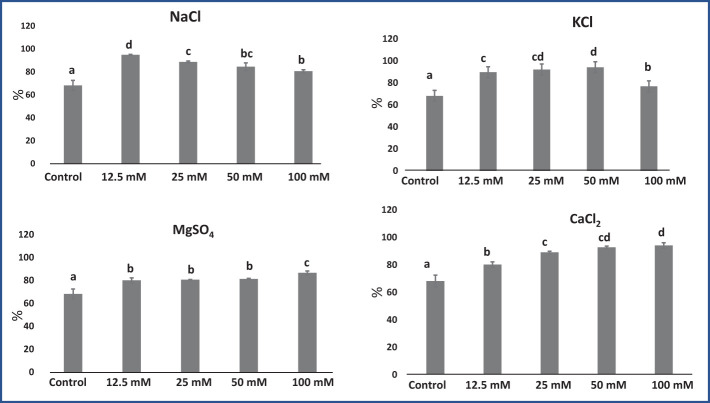
Effect of 12.5, 25, 50, and 100 mM concentrations of NaCl, KCl, MgSO_4_, and CaCl_2_ salts on electrolyte leakage (%) in an *M. longifolia* leaf sample. The data are represented as mean ± standard deviation (n = 3). The different letters on the bar graph show the significantly different groups according to the Duncan test (α = 0.05).

An El test demonstrates the leaf sample’s membrane stability (Henson et al., 1981). El increased significantly with increasing salt concentrations, as shown in **Figure 4**. On average, in *M. longifolia* leaves treated with various concentrations of different salts, the highest El was observed in CaCl_2_ (89.04%), followed by KCl (88.21%), NaCl (87.17%), and MgSO_4_ (82.10%). Plants in the control group had the lowest El (68.16%). Among the treatments with different concentrations of NaCl, El was highest at 12.5 mM and decreased with increasing NaCl concentrations. With KCl, El increased up to 50 mM of salt concentration, while it slightly decreased at 100 mM of concentration. Although, on treatment with MgSO_4_ and CaCl_2_, EI significantly increased with increasing salt concentrations.

### 3.3 Photosynthetic pigments

**Figure 5:**
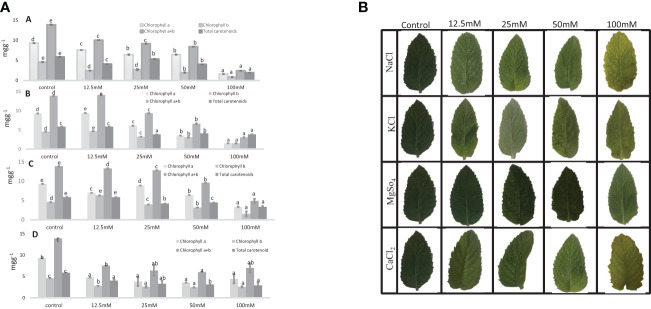
**1.** Chlorophyll content and total carotenoid content (mg g^−1^) in an *M. longifolia* leaf sample under salt stress condition. **(A)** Different concentrations of NaCl; **(B)** Different concentrations of KCl; **(C)** Different concentrations of MgSO_4_; and **(D)** Different concentrations of CaCl_2_. The data are represented as mean ± standard deviation (n = 3). The different letters on the same color bar show the significantly different groups according to the Duncan test (α = 0.05). **2.** Photosynthetic pigment in an *M. longifolia* leaf sample under different salt stress conditions.

In *M. longifolia* plants treated with different concentrations of salts, the levels of photosynthetic pigments were significantly decreased with an increasing salt concentration in the soil when compared to the control. The maximum reduction in pigments was noticed in all salt treatments at 100 mM concentration. The NaCl treatment showed the most detrimental effect on the content of pigments: chl a, chl b, chl a + b, and carotenoid (1.58, 0.84, 2.43, and 1.96 mg g^−1^) in comparison with the control plant (9.30, 4.58, 13.89, and 5.86 mg g^−1^), respectively (**Figure 5**). After NaCl, KCl showed the maximum reduction in pigment at 100 mM concentration. In KCl treatment, chl a was reduced from 9.39 mg g^−1^ (12.5 mM) to 1.58 mg g^−1^ (100 mM); chl b from 4.70 mg g^−1^ (12.5 mM) to 1.51 mg g^−1^ (100 mM); and chl a + b from 14.11 mg g^−1^ (12.5 mM) to 3.04 mg g^−1^ (100 mM) (**Figure 5**). The MgSO_4_ treatment resulted in the least negative effect on pigment content compared to other salt treatments (**Figure 5**).

### 3.4 Proline content

**Figure 6:**
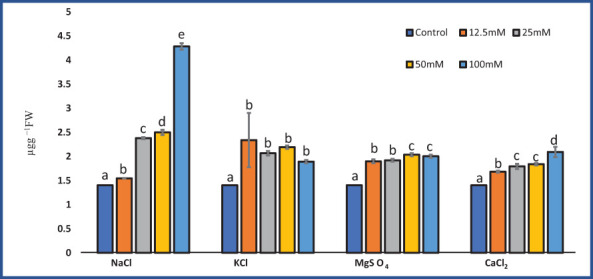
Effect of 12.5, 25, 50, and 100 mM concentrations of NaCl, KCl, MgSO_4_, and CaCl_2_ salts on proline content (µg g^−1^ fresh weight) in an *M. longifolia* leaf sample. The data are represented as mean ± standard deviation (n = 3). The different letters on the same color bar show the significantly different groups according to the Duncan test (α = 0.05).

In our experiments, the proline content in *M. longifolia* ranged between 1.40 µg g^−1^ (control) and 4.28 µg g^−1^ (100 mM NaCl). NaCl, MgSO_4_, and CaCl_2_ all had a significant effect on proline content (F = 26.62; p = 0.00), (F = 216.22; p = 0.00), and (F = 60.37; p = 0.00), respectively. While KCl treatment did not significantly (F = 6.13; p = 0.09) affect proline content. According to observations, the proline content increased with increasing salt concentration (**Figure 6**). When compared to all salt treatments, NaCl showed variability in proline content. Approximately 12.5 mM NaCl showed the lowest proline content (1.54 µg g^−1^) followed by 100 mM NaCl, which showed the highest proline content (4.28 µg g^−1^). In plants treated with 12.5 mM and 50 mM of KCl, the proline content was increased by 2.34 and 2.193 µg g^−1^), and moderately decreased when treated with 25 mM and 100 mM (2.06 and 1.89 µg g^−1^, respectively). The MgSO_4_ salt stress showed an increase in proline content (2.023 µg g^−1^) up to 50 mM and then slightly decreased (2.003 µg g^−1^) at 100 mM concentration.

### 3.5 DPPH inhibition

**Figure 7:**
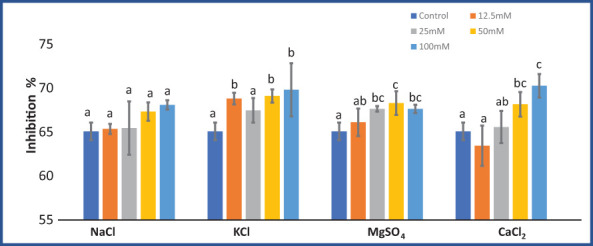
Effect of 12.5, 25, 50, and 100 mM concentrations of NaCl, KCl, MgSO_4_, and CaCl_2_ salts on DPPH inhibition % (antioxidant activity) in an *M. longifolia* leaf sample. The data are represented as mean ± standard deviation (n = 3). The different letters on the same color bar show the significantly different groups according to the Duncan test (α = 0.05).

KCl (F = 4.01; p = 0.03), MgSO_4_ (F = 4.79; p = 0.02), and CaCl_2_ (F = 8.28; p = 0.003) significantly affected the DPPH inhibition percentage. While NaCl salt treatment did not significantly (F = 2.31; p = 0.12) affect DPPH activity. DPPH inhibition in *M. longifolia* increased with increasing salt concentrations (**Figure 7**). At 100 mM salt concentrations, CaCl_2_ treatment resulted in the highest scavenging activity (70.26%), followed by KCl (69.80%), and NaCl (68.09%), whereas MgSO_4_-treated plants showed the least activity (67.63%). Instead of 12.5 mM CaCl_2_, all salt treatments led to an increase in scavenging activity as compared to the control group.

### 3.6 Essential oil yield

**Figure 8:**
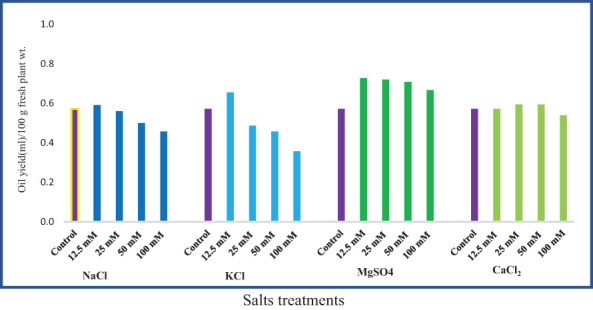
Effect of 12.5, 25, 50, and 100 mM concentrations of NaCl, KCl, MgSO_4_, and CaCl_2_ salts on essential oil yield in an *M. longifolia* leaf sample.

The essential oil yield of *M. longifolia* ranges from 0.357 ml (100 mM KCl) to 0.727 ml (12.5 mM MgSO_4_) in 100 g of fresh plant sample (**Figure 8**). Different concentrations of MgSO_4_ showed a positive effect on oil yield (27.27%, 26.00%, 23.95%, and 16.66%), respectively, compared to the control. Approximately 12.5 mM concentrations of NaCl and KCl caused an increment in oil yield of 3.42% and 14.65%, respectively, while the yield was decreased when NaCl and KCl concentrations were increased. In samples treated with 12.5, 25, and 50 mM of CaCl_2_, the oil yield was found to be increased by 0.71%, 4.05%, and 3.90%, respectively, compared to the control group.

### 3.7 Components of essential oil

**Figure 9:**
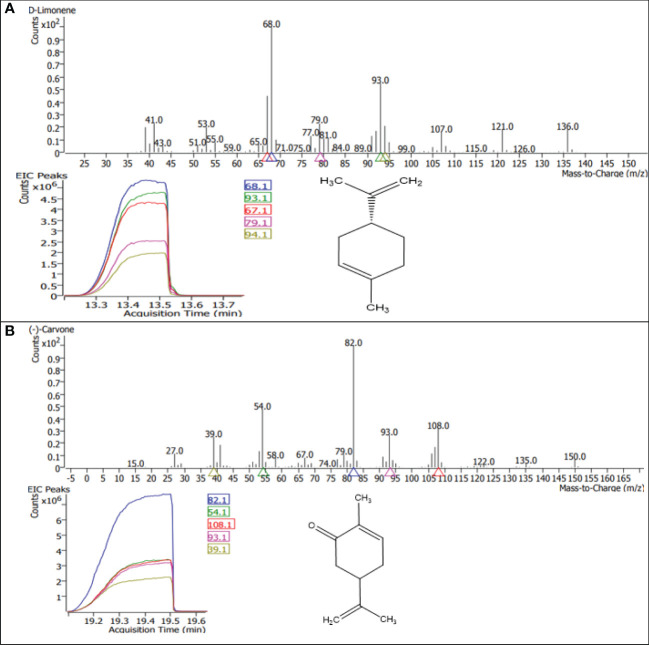
GC–MS chromatograms of the dominant compounds **(A)** limonene and **(B)** carvone of essential oil in *M. longifolia*.

**Figure 10:**
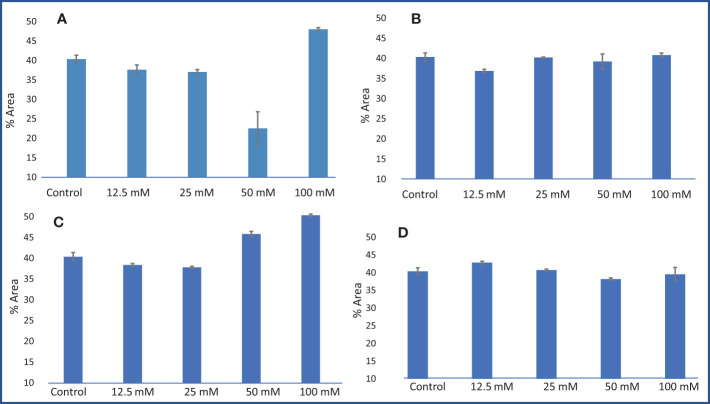
Limonene % area obtained from GC–MS analysis in an *M. longifolia* essential oil sample under salt stress condition. **(A)** Different concentrations of NaCl; **(B)** Different concentrations of KCl; **(C)** Different concentrations of MgSO_4_; and **(D)** Different concentrations of CaCl_2_. The data are represented as mean ± standard deviation (n = 3).

**Figure 11:**
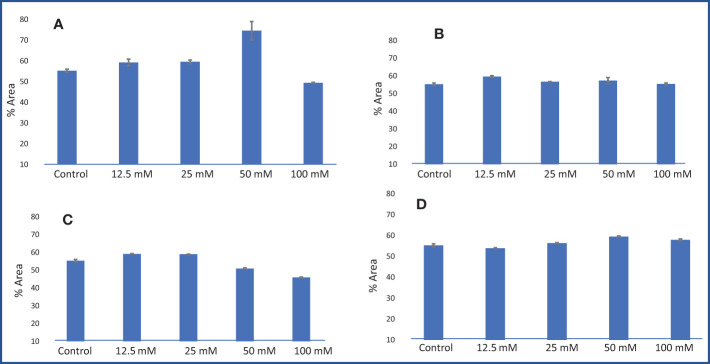
Carvone % area obtained from GC–MS analysis in an *M. longifolia* essential oil sample under salt stress condition. **(A)** Different concentrations of NaCl; **(B)** Different concentrations of KCl; **(C)** Different concentrations of MgSO_4_; and **(D)** Different concentrations of CaCl_2_. The data are represented as mean ± standard deviation (n = 3).

A gas chromatography–mass spectrometry (GC–MS) study of the composition of essential oils is provided in **Tables 4**–**7**. Salt stress significantly affected all the components identified in the oil of *M. longifolia*. The two main constituents of essential oils were (−)-carvone and D-limonene. These two components occupied 96% of the essential oils. The chromatogram and mass spectra of (−)-carvone and D-limonene from the control sample are shown in **Figure 9**. The samples treated with 50 mM CaCl_2_ showed the highest concentration of carvone (7.58%), which is higher than that of 100 mM CaCl_2_ (4.67%) as compared to the control. A negative relationship exists between the accumulation percentages of limonene and carvone in our experiments. Salt treatments such as NaCl and KCl (100 mM), MgSO_4_ (50 mM and 100 mM), and CaCl_2_ (12.5 mM) decreased the accumulation of carvone, whereas a significant increase in limonene content was seen at the above-mentioned concentrations (18.98%, 1.16%, 13.51%, 24.70%, 6.10%, and 0.86%, respectively) (**Figures 10**, **11**).

**Table 4:** Composition of essential oil under different NaCl salt concentration treatments of *M. longifolia* fresh plant material.

Compound name	CONTROL	12.5 mM NaCl	25 mM NaCl	50 mM NaCl	100 mM NaCl
alpha.-Pinene	0.201 ± 0.049a	0.249 ± 0.063a	0.214 ± 0.042ab	0.136 ± 0.038a	0.208 ± 0.015a–c
Camphene	0.013 ± 0.011a	0.014 ± 0.013a	0.02 ± 0.001a	0.032 ± 0.047a	0.105 ± 0.09a–c
3-methylene-6-(1-methylethyl)-cyclohexene	0.166 ± 0.037a	0.163 ± 0.036a	0.142 ± 0.023ab	0.07 ± 0.015a	0.15 ± 0.009a–c
beta.-Pinene	0.234 ± 0.219a	0.372 ± 0.126a	0.313 ± 0.059ab	0.13 ± 0.133a	0.314 ± 0.026cd
1,6-Octadiene, 7-methyl-3-methylene-	0.583 ± 0.244a	0.36 ± 0.361a	0.26 ± 0.229ab	0.08 ± 0.101a	0.46 ± 0.056d
D-Limonene	40.363 ± 0.998c	37.613 ± 1.25b	37.02 ± 0.637c	22.533 ± 4.29b	48.027 ± 0.428e
Eucalyptol	0.352 ± 0.610a	0.348 ± 0.419a	0.697 ± 0.604a	0.358 ± 0.337a	0 ± 0
1,6-Octadien-3-ol, 3,7-dimethyl-	0.021 ± 0.019a	0.145 ± 0.183a	0.031 ± 0.007a	0.023 ± 0.02a	0.033 ± 0.002a
endo-Borneol	0.089 ± 0.020b	0.07 ± 0.061a	0.037 ± 0.063a	0.088 ± 0.077a	0.079 ± 0.004a–c
Cyclohexanone, 2-methyl-5-(1-methylethenyl)-	1.627 ± 0.736a	0.416 ± 0.098a	0.367 ± 0.113ab	0.416 ± 0.052a	0.281 ± 0.024b–d
(−)-Carvone	55.317 ± 0.69d	59.323 ± 1.563c	59.627 ± 0.859d	74.663 ± 4.441c	49.477 ± 0.211f
(−)-8-p-Menthen-2-yl, acetate, trans	0.062 ± 0.031a	0.036 ± 0.012a	0.025 ± 0.011a	0.053 ± 0.018a	0.036 ± 0.003a
(−)-beta-Bourbonene	0.071 ± 0.029a	0.044 ± 0.012a	0.032 ± 0.011a	0.054 ± 0.016a	0.046 ± 0.002ab
Caryophyllene	0.264 ± 0.144a	0.088 ± 0.124a	0.078 ± 0.135a	0.217 ± 0.195a	0.167 ± 0.016a–c
cis-beta-Farnesene	0.03 ± 0.035a	0.019 ± 0.018a	0.022 ± 0.019a	0.013 ± 0.022a	0.101 ± 0.132a–c
1,6,10-Dodecatriene, 7,11-dimethyl-3-methylene-	0.021 ± 0.018a	0.03 ± 0.028a	0.018 ± 0.017a	0.018 ± 0.031a	0 ± 0
8-isopropyl-1-methyl-5-methylene-1,6-cyclodecadiene	0.028 ± 0.048a	0.09 ± 0.029a	0.085 ± 0.054a	0.14 ± 0.054a	0.075 ± 0.005ab

Means in the same column by the same letter are not significantly different to the test of Duncan (α = 0.05).

**Table 5:** Composition of essential oil under different KCl salt concentration treatments of *M. longifolia* fresh plant material.

Compound name	CONTROL	12.5 mM KCl	25 mM KCl	50 mM KCl	100 mM KCl
alpha.-Pinene	0.201 ± 0.049a	0.17 ± 0.006ab	0.857 ± 0.569c	0.518 ± 0.582a-c	0.165 ± 0.001c
Camphene	0.013 ± 0.011a	0.019 ± 0.001a	0.02 ± 0a	0.02 ± 0ab	0.021 ± 0.002a
3-methylene-6-(1-methylethyl)-cyclohexene	0.166 ± 0.037a	0.133 ± 0.004ab	0.142 ± 0.005a	0.134 ± 0.014a-c	0.134 ± 0.024bc
beta.-Pinene	0.234 ± 0.219a	0.17 ± 0.147ab	0.286 ± 0.013ab	0.186 ± 0.152c	0.122 ± 0.191d
1,6-Octadiene, 7-methyl-3-methylene-	0.583 ± 0.244a	0.434 ± 0.011ab	0.436 ± 0.041b	0.435 ± 0.052d	0.369 ± 0.01f
D-Limonene	40.363 ± 0.998c	36.87 ± 0.416bc	40.2 ± 0.137d	39.253 ± 1.856e	40.833 ± 0.495h
Eucalyptol	0.352 ± 0.610a	0.915 ± 0.038e	0.912 ± 0.048c	0.906 ± 0.027a	0.918 ± 0.039a
1,6-Octadien-3-ol, 3,7-dimethyl-	0.021 ± 0.019a	0.027 ± 0.002d	0.027 ± 0.002a	0.024 ± 0.003ab	0.019 ± 0.008ab
endo-Borneol	0.089 ± 0.020b	0.058 ± 0.05a	0.051 ± 0.044a	0.056 ± 0.049ab	0.086 ± 0.006ab
Cyclohexanone, 2-methyl-5-(1-methylethenyl)-	1.627 ± 0.736a	0.655 ± 0.002cd	0.149 ± 0.187a	0.34 ± 0.262bc	0.593 ± 0.007e
(−)-Carvone	55.317 ± 0.69d	59.64 ± 0.468f	56.7 ± 0.105e	57.37 ± 1.745f	55.46 ± 0.614g
(−)-8-p-Menthen-2-yl, acetate, trans	0.062 ± 0.031a	0.057 ± 0.001a	0.029 ± 0.003a	0.052 ± 0.022ab	0.081 ± 0.01ab
(−)-beta-Bourbonene	0.071 ± 0.029a	0.045 ± 0.001a	0.038 ± 0.001a	0.049 ± 0.015ab	0.072 ± 0.008ab
Caryophyllene	0.264 ± 0.144a	0.425 ± 0.404bc	0.14 ± 0.012a	0.167 ± 0.035a–c	0.153 ± 0.063ab
cis-beta-Farnesene	0.03 ± 0.035a	0.033 ± 0.001a	0.022 ± 0.001a	0.03 ± 0.008a	0.137 ± 0.173a
1,6,10-Dodecatriene, 7,11-dimethyl-3-methylene-	0.021 ± 0.018a	0 ± 0	0 ± 0	0 ± 0	0 ± 0
8-isopropyl-1-methyl-5-methylene-1,6-cyclodecadiene	0.028 ± 0.048a	0.104 ± 0.001ab	0.055 ± 0.005a	0.061 ± 0.013ab	0.08 ± 0.006ab

Means in the same column by the same letter are not significantly different to the test of Duncan (α = 0.05).

**Table 6:** Composition of essential oil under different MgSO_4_ salt concentration treatments of *M. longifolia* fresh plant material.

Compound name	CONTROL	12.5 mM MgSO_4_	25 mM MgSO_4_	50 mM MgSO_4_	100 mM MgSO_4_
alpha.-Pinene	0.201 ± 0.049a	0.187 ± 0.007bc	0.192 ± 0.004a	0.229 ± 0.171a–c	0.298 ± 0.008c
Camphene	0.013 ± 0.011a	0.019 ± 0.001ab	0.019 ± 0a	0.116 ± 0.15ab	0.028 ± 0.001a
3-methylene-6-(1-methylethyl)-cyclohexene	0.166 ± 0.037a	0.133 ± 0.004a–c	0.142 ± 0.003a	0.221 ± 0.008a–c	0.202 ± 0.004bc
beta.-Pinene	0.234 ± 0.219a	0.274 ± 0.007cd	0.095 ± 0.165a	0.499 ± 0.034c	0.482 ± 0.009d
1,6-Octadiene, 7-methyl-3-methylene-	0.583 ± 0.244a	0.467 ± 0.006e	0.519 ± 0.017bc	0.843 ± 0.051d	0.95 ± 0.021f
D-Limonene	40.363 ± 0.998c	38.357 ± 0.341f	37.797 ± 0.239d	45.82 ± 0.64e	50.333 ± 0.263h
Eucalyptol	0.352 ± 0.610a	0 ± 0	0.328 ± 0.492ab	0 ± 0	0 ± 0
1,6-Octadien-3-ol, 3,7-dimethyl-	0.021 ± 0.019a	0.027 ± 0.001ab	0.031 ± 0.002a	0.037 ± 0.002ab	0.042 ± 0ab
endo-Borneol	0.089 ± 0.020b	0.085 ± 0.002ab	0.087 ± 0.004a	0.093 ± 0.006ab	0.08 ± 0.002ab
Cyclohexanone, 2-methyl-5-(1-methylethenyl)	1.627 ± 0.736a	0.402 ± 0.009de	0.674 ± 0.037c	0.372 ± 0.044bc	0.787 ± 0.026e
(−)-Carvone	55.317 ± 0.69d	59.15 ± 0.156g	58.947 ± 0.145e	50.937 ± 0.366f	45.903 ± 0.189g
(−)-8-p-Menthen-2-yl, acetate, trans	0.062 ± 0.031a	0.057 ± 0.003ab	0.07 ± 0.006a	0.046 ± 0.007ab	0.064 ± 0.003ab
(−)-beta-Bourbonene	0.071 ± 0.029a	0.048 ± 0.001ab	0.207 ± 0.261a	0.04 ± 0.006ab	0.057 ± 0.002ab
Caryophyllene	0.264 ± 0.144a	0.187 ± 0.013bc	0.174 ± 0.151a	0.166 ± 0.026a–c	0.141 ± 0.123ab
cis-beta-Farnesene	0.03 ± 0.035a	0.023 ± 0.02ab	0.013 ± 0.023a	0 ± 0	0.031 ± 0a
1,6,10-Dodecatriene, 7,11-dimethyl-3-methylene	0.021 ± 0.018a	0.009 ± 0.008ab	0.026 ± 0.023a	0.025 ± 0.003ab	0 ± 0
8-isopropyl-1-methyl-5-methylene-1,6-cyclodecadiene	0.028 ± 0.048a	0.079 ± 0.002ab	0.129 ± 0.01a	0.072 ± 0.012ab	0.08 ± 0.002ab

Means in the same column by the same letter are not significantly different to the test of Duncan (α = 0.05).

**Table 7:** Composition of essential oil under different CaCl_2_ salt concentration treatments of *M. longifolia* fresh plant material.

Compound name	CONTROL	12.5 mM CaCl_2_	25 mM CaCl_2_	50 mM CaCl_2_	100 mM CaCl_2_
alpha.-Pinene	0.201 ± 0.049a	0.289 ± 0.002bc	0.233 ± 0.002c–e	0.112 ± 0.083ab	0.185 ± 0.019ab
Camphene	0.013 ± 0.011a	0.026 ± 0a	0.021 ± 0ab	0.018 ± 0a	0.079 ± 0.101a
3-methylene-6-(1-methylethyl)-cyclohexene	0.166 ± 0.037a	0.205 ± 0.003ab	0.161 ± 0.005bd	0.158 ± 0.003ab	0.164 ± 0.018ab
beta.-Pinene	0.234 ± 0.219a	0.436 ± 0.004c	0.342 ± 0.002e	0.293 ± 0.011bc	0.304 ± 0.045ab
1,6-Octadiene, 7-methyl-3-methylene-	0.583 ± 0.244a	0.841 ± 0.018d	0.676 ± 0.006f	0.563 ± 0.027d	0.534 ± 0.126b
D-Limonene	40.363 ± 0.998c	42.827 ± 0.348e	40.713 ± 0.237g	38.17 ± 0.312e	39.513 ± 0.195c
Eucalyptol	0.352 ± 0.610a	0.002 ± 0.004a	0 ± 0	0 ± 0	0.378 ± 0.655ab
1,6-Octadien-3-ol, 3,7-dimethyl-	0.021 ± 0.019a	0.043 ± 0.003a	0.033 ± 0.003ab	0.047 ± 0.003a	0.04 ± 0.006a
endo-Borneol	0.089 ± 0.020b	0.112 ± 0.002ab	0.093 ± 0.001ac	0.12 ± 0.004ab	0.074 ± 0.064a
Cyclohexanone, 2-methyl-5-(1-methylethenyl)-	1.627 ± 0.736a	0.425 ± 0.02c	0.373 ± 0.005e	0.436 ± 0.025cd	0.233 ± 0.062ab
(−)-Carvone	55.317 ± 0.69d	53.93 ± 0.255f	56.35 ± 0.242h	59.513 ± 0.309f	57.903 ± 0.411d
(−)-8-p-Menthen-2-yl, acetate, trans	0.062 ± 0.031a	0.032 ± 0.002a	0.044 ± 0.001ab	0.044 ± 0.002a	0.026 ± 0.009a
(−)-beta-Bourbonene	0.071 ± 0.029a	0.066 ± 0.004a	0.065 ± 0ab	0.027 ± 0.003a	0.043 ± 0.01a
Caryophyllene	0.264 ± 0.144a	0.292 ± 0.025bc	0.295 ± 0.002de	0.068 ± 0.059a	0.385 ± 0.533ab
cis-beta-Farnesene	0.03 ± 0.035a	0.024 ± 0.021a	0 ± 0	0.012 ± 0.01a	0.006 ± 0.01a
1,6,10-Dodecatriene, 7,11-dimethyl-3-methylene-	0.021 ± 0.018a	0.013 ± 0.022a	0.041 ± 0.001ab	0.006 ± 0.01a	0.02 ± 0.018a
8-isopropyl-1-methyl-5-methylene-1,6-cyclodecadiene	0.028 ± 0.048a	0.114 ± 0.01ab	0.118 ± 0ac	0.058 ± 0.026a	0.068 ± 0.019a

Means in the same column by the same letter are not significantly different to the test of Duncan (α = 0.05).

Other components obtained from the essential oil of *M. longifolia* with more than 0.2% in the control plant group were alpha-pinene (0.20%), beta-pinene (0.23%), eucalyptol (0.35%), cyclohexanone, 2-methyl-5-1-methylethenyl (1.62%), and caryophyllene (0.26%) (**Tables 4**-**7**).

### 3.8 Correlation analysis and principal component (PCA) analysis

**Figure 12:**
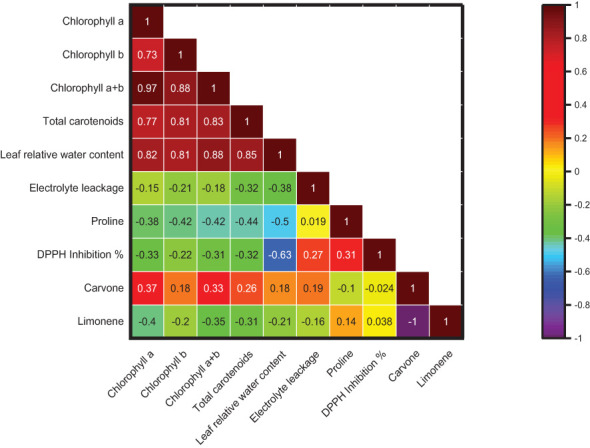
Correlation among chlorophyll (a), chlorophyll (b), chlorophyll (a + b), total carotenoids, leaf relative water content, electrolyte leakage, proline content, DPPH inhibition %, carvone and limonene % area. The brown color boxes show a strong correlation, and the purple color boxes show a weak correlation.

The Pearson correlation was plotted among different plants’ physiological and biochemical parameters, such as photosynthetic pigment, El, RWC, proline content, DPPH inhibition %, carvone, and limonene %, with different salt stress treatments in *M. longifolia* (**Figure 12**). According to the observation, the percentages of photosynthetic pigment, RWC, and carvone were negatively correlated with the percentages of El, proline content, DPPH inhibition, and limonene. However, the pigments have a strong positive correlation with each other, represented by brown color, and show a high correlation coefficient (r) value (r = 0.73 to r = 0.97). Moreover, the carvone and limonene percentages show a strong negative correlation (r = −1), represented by the purple color.

**Figure 13:**
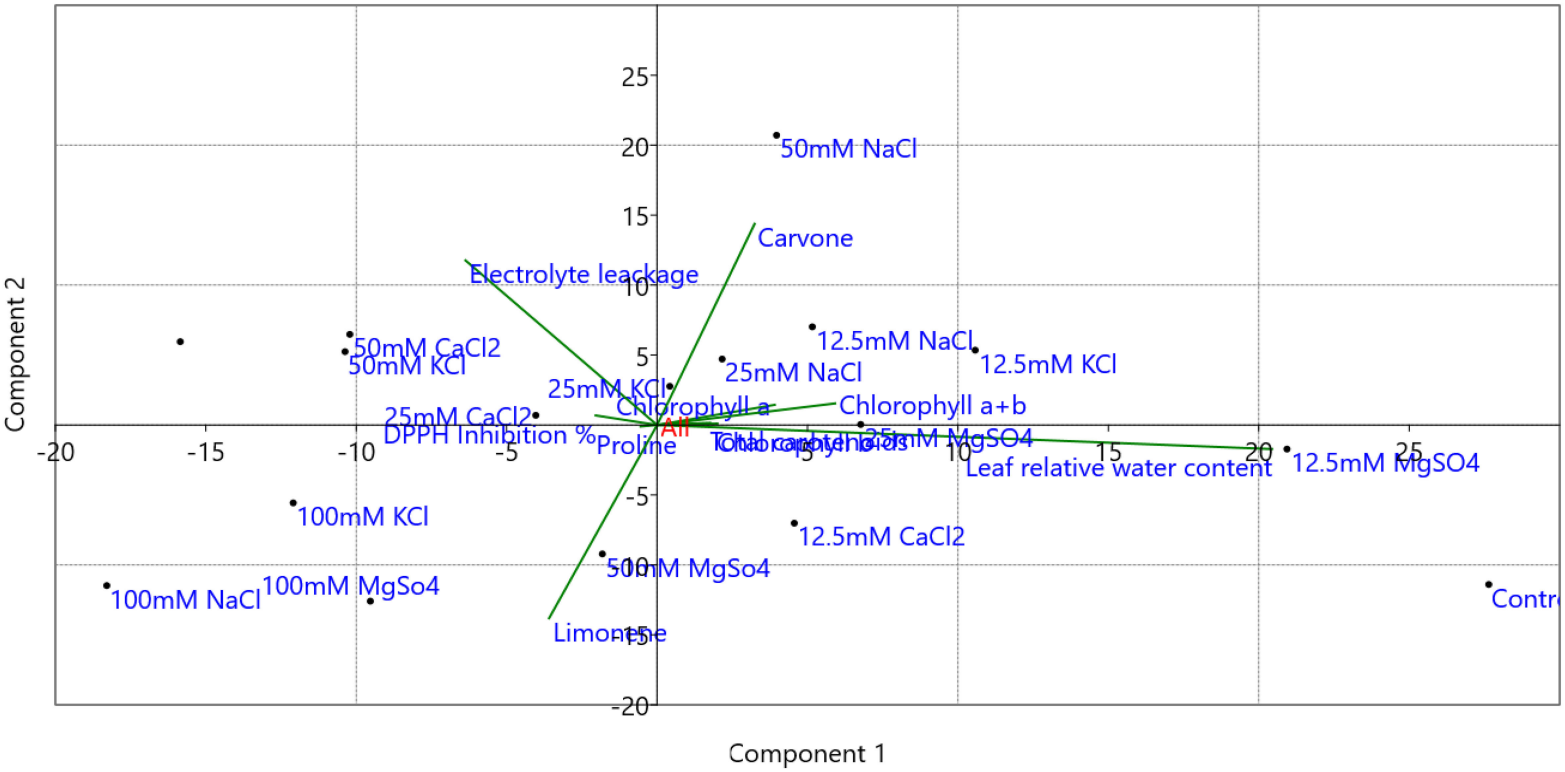
Principal component analysis (PCA) biplots of *M. longifolia* chlorophyll (a), chlorophyll (b), chlorophyll (a + b), total carotenoids, leaf relative water content, electrolyte leakage, proline content, DPPH inhibition %, carvone and limonene % area with different (12.5, 25, 50, and 100 mM) concentrations of NaCl, KCl, MgSO_4_, and CaCl_2_ salts compared to the control plant.

The physico-chemical parameters such as photosynthetic pigments, El, RWC, proline content, DPPH inhibition percentage, carvone, and limonene percentage were subjected to principal component analysis (**Figure 13**). The biplot (score and loading) from PCA displayed that the first two PCs exhibited overall variability of 84.50%. However, the second and third PCs showed overall variability of 97.48%. The PC1 independently has 56.50% variance, and the PC2 independently has 28.0% variance. The pigments and percentage of carvone have a positive association with PC1 and PC2. The El and DPPH percentage inhibition have a positive association with PC1 and a negative association with PC2, whereas the proline content and limonene percentage have a negative association with PC1 and PC2.

### 3.9 Expression analysis of key genes involved in carvone and limonene biosynthesis

**Figure 14:**
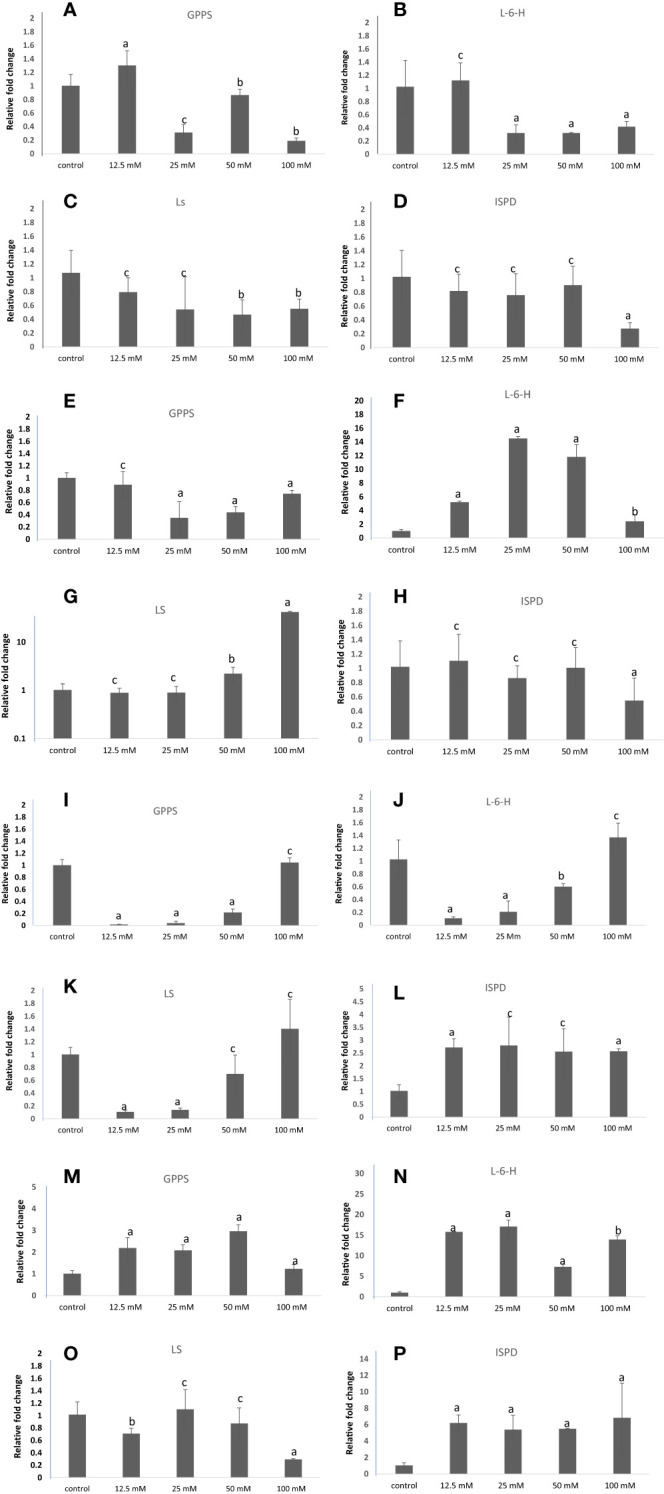
qRT-PCR-based expression analysis of key genes of the carvone and limonene biosynthesis pathways of *M. longifolia*. The bar diagrams represent the fold change in expression of each gene upon 12.5, 25, 50, and 100 mM salt treatment as compared to untreated controls. **(A–D)** Different concentrations of NaCl; **(E–H)** Different concentrations of KCl; **(I–L)** Different concentrations of MgSO_4_; and **(M–P)** Different concentrations of CaCl_2_. The results are presented as the means of three replicates and as means ± SDs. Statistical significance was determined by the Student’s t-test. Letters a and b denote the significance of fold changes at p-values <0.05 (a <0.005, b<0.05) while letter c denotes the non-significant nature of fold changes at p-values >0.05.

Geranyl pyrophosphate synthase (GPPS), Limonene-6-hydroxylase (L-6-H), Limonene synthase (LS), and Isopiperitenol dehydrogenase (ISPD) are the key genes involved in the carvone and limonene biosynthetic pathways. Their expression was examined at the transcript level in samples treated with different concentrations of NaCl, KCl, MgSO_4_, and CaCl_2_ (**Figure 14**). These genes showed significant changes in their expression patterns in treated plants compared to the control group of *M. longifolia*. As compared to control, the expression of GPPS and L-6-H was increased by 1.30 and 1.12 folds with 12.5 mM NaCl treatment, respectively, and decreased (1.13, 0.81, and 0.18 folds, respectively) with 25, 50, and 100 mM NaCl treatment. With 12.5, 25, and 50 mM NaCl treatment, LS gene expression decreased by 0.09, 0.64, and 0.55-fold, and slightly increased by 0.65-fold with 100 mM NaCl but did not exceed control levels. At 50 mM NaCl treatment, there was no significant variation in the expression of the ISPD gene. But it significantly decreased with 100 mM NaCl treatment compared to control.

GPPS expression was not significantly altered with 12.5 mM of KCl concentration, but was significantly decreased (0.34, 0.43, and 0.74 folds) with 25, 50, and 100 mM KCl concentrations, respectively, when compared with control. Moreover, the expression level of the L-6-H gene was found to be significantly increased by 14.50-fold in plants exposed to 25 mM of KCl and then further decreased (11.28 and 2.41-fold) with 50 mM and 100 mM of KCl, but not less than control. With 25 and 50 mM of KCl treatment, the expression of the LS gene did not significantly change, but with 50 and 100 mM of KCl, it increased substantially by 2.22 and 41.70-fold, respectively. 12.5, 25, and 50 mM KCl treatments did not significantly affect the expression of the ISPD gene, while 100 mM KCl treatments significantly decreased it compared with the control.

On exposure to 12.5, 25, and 50 mM MgSO_4_, the expression of the GPPS and L-6-H genes decreased by 0.01, 0.04, 0.21-fold, and 0.10, 0.20, and 0.60-fold, respectively, and increased by 1.04-fold and 1.3-fold with 100 mM MgSO_4_ treatment. Similarly, MgSO_4_ at 12.5 and 25 mM decreased the transcripts of the LS gene. In comparison to the control plant group, the transcript level of ISPD increased at all concentrations of MgSO_4_. As compared with the control, the CaCl_2_ salt increased the transcription levels of the GPPS and L-6-H genes. Furthermore, the LS gene expression slightly decreased at 12.5 mM CaCl_2_, and no significant difference was observed at 25 and 100 mM CaCl_2_ concentrations. However, the expression of the ISPD gene increased by 6.19, 5.80, 5.47, and 6.82-folds with increasing concentrations of CaCl_2_ salt when compared to control.

## 4 Discussion

Salinity inhibits plant growth by causing osmotic stress, ionic imbalance, and nutritional imbalance (Khare et al., 2020; Ali et al., 2021; Ivy et al., 2022). Such deficits have a negative impact on plant growth and development (Shrivastava and Kumar, 2015). The current study was aimed at investigating the morphological, physiological, and biochemical status of *M. longifolia* exposed to four different types of salts (NaCl, CaCl_2_, KCl, and MgSO_4_) at four different concentrations (12.5, 25, 50, and 100 mM) in soil. The salt stress showed a detrimental effect on plant physiological and morphological parameters. The results revealed that NaCl, CaCl_2_, and KCl salt showed harmful effects at over 50 mM concentrations in *M. longifolia*. NaCl and KCl have the most toxic effects at higher concentrations than CaCl_2_ and MgSO_4_. Similarly, Kulak et al. (2020) also noted that NaCl and KCl are more detrimental than CaCl_2_ and MgSO_4_ to *S. officinalis*. Interestingly, among these salts, 12.5 mM, and 25 mM of MgSO_4_ have a beneficial effect on plant height. However, the biomass was found to be reduced in plants that grew at all concentrations of MgSO_4_.

The effects of salinity caused by NaCl on plant physiology are common (Fageria et al., 2012). The previous study also shows that increased concentrations of NaCl have an adverse effect on morphology in other plants, such as *Dracocephalum moldavia* L. (Mohammadi et al., 2021), *Ocimum basilicum* L. (Lazarević et al., 2021), *Oenanthe javanica* (Kumar et al., 2021), *Suaeda salsa* (Zhang et al., 2020), *Glycine max* L. (Jabeen et al., 2021), *Hordeum vulgare*, *Lactuca sativa*, and *Helianthus annuus* (Szymańska et al., 2022). High Na^+^ interferes with the allocation of other important minerals and reduces water uptake; it alters membrane lipids and induces ROS generation, leading to lipid peroxidation, resulting in a decrease in RWC, photosynthetic pigments, and an increase in El, proline content, DPPH activity in *M. longifolia.* High Na^+^ levels can depolarize and injure the plasma membrane, limiting K^+^ uptake and leaking; additionally, Na^+^ can replace K^+^ in key enzymatic reactions, further compromising cellular functions in the cytosol and organelles (Almeida et al., 2017; Cirillo et al., 2019). A similar study was also documented in *Dracoceplalum kotschyi*, where the increasing NaCl (25, 50, 75, and 100 mM) concentration decreased the RWC and photosynthetic pigments had increased El and proline content (Vafadar et al., 2020). In *Thymus vulgaris* and *Thymus daenensis*, the application of NaCl stress reduced the chlorophyll content and seedling growth and increased the El (Bistgani et al., 2019). In *O. basilicum*, the membrane permeability, essential oil content, and proline content were increased with NaCl salt stress (Farsaraei et al., 2020). Salt stress lowers the rate of photosynthesis. This might also be a direct result of the salt-induced leaf lamina width reduction; in fact, the area-based photosynthetic capacity is directly related to leaf width (Wyka et al., 2012). Osmotic stress can also adversely affect the activities of stomatal enzymes and the closure of stomata, which in turn results in CO_2_ reduction. Salt stress also inhibits the electron transport chain and results in the inactivation of photosystem II reaction centers. It also destroys the oxygen evolution complex (OEC) and impairs the electron transfer capacity on the donor side of PSII. Increased levels of sodium ions in the non-stomatal leaf tissues can also significantly affect the metabolic processes that limit photosynthesis. Houimli et al. (2010) reported that NaCl stress has more effects on chlorophyll b than chlorophyll a. This increase in the chlorophyll a/chlorophyll b ratio in NaCl exposure is because of the first step of chlorophyll b degradation that results in its conversion into chlorophyll a, and therefore, a decrease in total chlorophyll content (Gomes et al., 2017).

KCl has the same salty taste as NaCl and is characterized by relatively offensive side tastes of acridity, metallicity, and bitterness (Rasheed et al., 2020). Likewise, NaCl and KCl also significantly reduce the plants’ morphological attributes, although the reduction in plants’ height and number of branches was not correlated linearly with increasing concentrations. Previously, it was reported that treatment with 300 mM KCl in *Chenopodium album* reduced growth and significantly reduced carotenoid levels (Yao et al., 2010). With rising concentrations of 50, 100, and 150 mM of KCl salt, the root and shoot growth of *Carthamus tinctorius* L. was also reduced (Leblebİcİ et al., 2021). The growth of *Triticum aestivum* L. was reduced by more than 60 ppm of KCl (Rasheed et al., 2020). When KCl is available in the soil, the plant experiences an overload of K^+^ accumulation in the cytoplasm, which destroys the K^+^/Na^+^ homeostasis and causes ion toxicity. Further, high KCl also causes the physiological drought condition in plants, causing osmotic stress, which leads to decreases in chlorophyll content and RWC and increases in EL, proline content, and DPPH activity in *M. longifolia*. According to previous research, the chlorophyll content decreases with KCl treatment in *Malus hupehensis* and is recovered by the application of exogenous strigolactones (Ma et al., 2022), whereas the proline content increases with 50 mM and 75 mM KCl treatment in pomegranate varieties (Dichala et al., 2022).

The present study suggests that *M. longifolia* can withstand high MgSO_4_ concentrations (100 mM), and the negative impact of excess salt is as prominent as in the case of other salts such as NaCl, KCl, and CaCl_2_. Like Na^+^ and K^+^, the excessive concentration of Mg^2+^ in soil also alters the ion balance in the cytoplasm and affects several biosynthetic processes, resulting in a decrease in photosynthetic pigment and RWC in *M. longifolia.* Magnesium (Mg^2+^) is an important plant nutrient involved in a variety of physiological processes, and its deficiency can harm crop quality and yield (Jezek et al., 2015). Mg^2+^ has an impact on several metabolic processes and reactions, such as photophosphorylation (which results in ATP synthesis in chloroplasts), photosynthesis-based fixing of carbon dioxide (CO_2_), protein formation, chlorophyll development, phloem loading, photoassimilate partitioning and consumption, the production of reactive oxygen species, and photooxidation in leaf tissues (Tang et al., 2012). But excessive amounts of Mg^2+^ in soil result in Mg^2+^ toxicity in plants. Serpentine soil is a rich source of magnesium because of weathering ultramafic rocks, which contain at least 70% ferromagnesian composition. High levels of soil Mg^2+^ inhibit the uptake of Ca^2+^ by the plant (Brady et al., 2005). The growth of *Echinochloa* species was also reduced by excess MgCl_2_ or MgSO_4_ in the culture solution (30 mM), leading to symptoms like calcium deficiency. However, rice cultivars grew only 54%–67% slower than the control, and excess Mg^2+^ in the culture solution did not mimic the symptoms of Ca^2+^ deficiency (Kobayashi et al., 2005). Additionally, magnesium overdose caused coppery coloration and defoliation during the final stages of toxicity (Selvaraj and Sankar, 2010).

The Mg^2+^ concentration of 1.5 mM is considered optimal for *Arabidopsis* growth, and its lower or higher concentrations can interfere with numerous reaction processes, including ion uptake, starch production, and degradation, and it might be involved in reorganizing plant morphology (Guo et al., 2014). In leaves, Mg^2+^ toxicity caused overly rapid starch and sugar breakdown. Starch biosynthesis enzymes like SS1, SS2, GBSS1, and APL1 were significantly suppressed by Mg^2+^ toxicity, but AtAMY1 and BAM1 were significantly increased. Several genes that code for the phytohormone biosynthetic enzymes are significantly increased by magnesium toxicity, such as ethylene (1-aminocyclopropane-1-carboxylate synthase), jasmonic acid (lipoxygenase), and ABA (9-cis-epoxycarotenoid dioxygenase) (Guo et al., 2015).

Approximately 12.5 mM CaCl_2_ has a beneficial effect on plant height in *M. longifolia*. Although its increasing concentrations of 25, 50, and 100 mM hampered the growth and development of *Mentha*. The symplastic pathway has been shown to increase chlorine transport at high salinities (Geilfus, 2018). According to previous documentation, CaCl_2_-treated *Callistemon citrinus* reduces height, dry weight, and number of leaves (25.4%, 33.2%, and 60.7%, respectively) in comparison to non-salinized control plants. Moreover, in *Viburnum lucidum*, the nutrient solution with 53.33 mM CaCl_2_ is also responsible for the reduction in plant height, the number of leaves per plant, shoot dry biomass, and the relative growth compared to the control plant (Cirillo et al., 2019). CaCl_2_ significantly reduces canopy volume, total leaf area per plant, leaf dry weight, and plant height in *C. citrinus* (De Micco et al., 2021). The extra Ca^2+^, which is unlikely to create toxicity, can restrict the intake or accessibility of other minerals such as K^+^ in *V. lucidum* or PO4 ^3−^ in *C. citrinus*, contributing to a restrained relative growth rate (RGR) (White and Broadley, 2003). Based on our data, like NaCl and KCl, moderate to high CaCl_2_ levels in the soil create an adverse impact on photosynthetic pigment content and RWC but also increase the El, proline content, and DPPH scavenging activity.

Volatile compounds are synthesized in response to various environmental factors, such as soil minerals, light intensity, climatic conditions, and soil salinity. In aromatic plants, these factors affect oil yields and composition differently (Çoban and Baydar, 2016). The EO production in *M. longifolia* was greatly reduced with 100 mM NaCl, KCl, and CaCl_2_, respectively. This may be because of the reduction in photosynthetic pigment, which leads to lesser photosynthate assimilation, resulting in decreased biosynthesis of EO. A similar study documented that irrigation with 100 mM NaCl reduced EO yield in *Salvia* (Taarit et al., 2009); 150 mM NaCl treatment reduced EO in *Mentha piperita* L. (Çoban and Baydar, 2016); and in *Lallementia iberica* (Paravar et al., 2022).

However, the low concentrations (12.5 mM) of NaCl, KCl, MgSO_4_, and CaCl_2_ led to increased essential oil yields in *M. longifolia*. This may be because the low concentrations of these salts’ ions support the mycorrhizal association and humic substances, which benefit root ramification and improve water and phosphorous uptake (Hazzoumi et al., 2015). Moreover, it is possible to think of oil yield augmentation at moderate salt concentrations as a salinity adaptation feature. Free volatiles are glycosylated and kept in cell vacuoles. These contribute to cellular expansion, which lessens the impact of the osmotic stress brought on by salt (Biswas et al., 2011). According to an essential oil GC–MS analysis of *M. longifolia*, it was found that carvone and limonene are the most dominant compounds. The percentage of both compounds is highly variable with different salt treatments. A carvone is produced biochemically in three steps: geranyl pyrophosphate is converted into (−)- limonene by limonene synthase, which is then hydroxylated into (−)-trans carveol by limonene-6-hydroxylase, which is then dehydrated by isopiperitenol dehydrogenase into (−)-carvone. (Bouwmeester et al., 1998). The biosynthesis of carvone and limonene is interrelated as limonene is the precursor of carvone (Delfine et al., 2022). KCl, NaCl, and MgSO_4_ at high concentrations inhibit limonene’s conversion to carvone, while NaCl at 50 mM supports limonene’s conversion to carvone at its best.

Including all morphological and physicochemical findings, it appears that salinity may adversely affect water uptake, membrane integrity, and biochemical pathways, resulting in a decrease in growth and biomass. Additionally, salinity results in nutritional imbalance, excessive ROS formation, and inhibition of enzymatic activities, all of which have a negative impact on biological membranes and cellular components and lower biomass output (Alzahrani et al., 2019; Kumar et al., 2021).

## 5 Conclusion

Salinity stress decreased FW, DW, RWC, and pigment content but increased accumulation of essential oil, especially its main components (carvone and D-limonene), EL and DPPH activity of *M. longifolia* plants. Salt concentrations of 12.5 mM and 25 mM stimulated production of essential oils, especially carvone and D-limonene. It may be concluded that *M. longifolia* is tolerant of mild soil salinity; thus, we may recommend its cultivation in saline soils. Future studies may be taken to further understand the molecular basis for increased essential oil accumulation in saline environments, including deciphering the regulatory networks.

## Data availability statement

The raw data supporting the conclusions of this article will be made available by the authors, without undue reservation.

## Author contributions

RS carried out most of the experimental work and analysis as well as wrote the manuscript and prepared the figures. SA helped RS in carrying out molecular analysis of this research and manuscript writing. SL and GR helped RS in carrying out experimental works and preparation of manuscript. AG carried out GC–MS/MS analysis. SG and RB designed the study, supervised RB, SA, SL, and GR also proofread the manuscript and figures. All authors contributed to the article and approved the submitted version.

## References

[B1] Ahmed S. Asgher M. Kumar A. Gandhi S. G. (2022). Exogenously applied rohitukine inhibits photosynthetic processes, growth and induces antioxidant defense system in arabidopsis thaliana. Antioxidants 11, 1512. doi: 10.3390/antiox11081512 36009231 PMC9404761

[B2] Ali M. Kamran M. Abbasi G. H. Saleem M. H. Ahmad S. Parveen A.. (2021). Melatonin-induced salinity tolerance by ameliorating osmotic and oxidative stress in the seedlings of two tomato (Solanum lycopersicum l.) cultivars. J. Plant Growth Regul. 40, 2236–2248. doi: 10.1007/s00344-020-10273-3

[B3] Almeida D. M. Oliveira M. M. Saibo N. J. (2017). Regulation of na+ and k+ homeostasis in plants: towards improved salt stress tolerance in crop plants. Genet. Mol. Biol. 40, 326–345. doi: 10.1590/1678-4685-GMB-2016-0106 28350038 PMC5452131

[B4] Alzahrani S. M. Alaraidh I. A. Migdadi H. Alghamdi S. Khan M. A. Ahmad P. (2019). Physiological, biochemical, and antioxidant properties of two genotypes of vicia faba grown under salinity stress. Pak. J. Bot. 51, 786–798. doi: 10.30848/PJB2019-3(3

[B5] Araghi A. M. Nemati H. Azizi M. Moshtaghi N. Shoor M. Hadian J. (2019). Assessment of phytochemical and agro-morphological variability among different wild accessions of mentha longifolia l. cultivated in field condition. Ind. Crops Prod 140, 111698. doi: 10.1016/j.indcrop.2019.111698

[B6] Arif Y. Singh P. Siddiqui H. Bajguz A. Hayat S. (2020). Salinity induced physiological and biochemical changes in plants: An omic approach towards salt stress tolerance. Plant Physiol. Biochem. 156, 64–77. doi: 10.1016/j.plaphy.2020.08.042 32906023

[B7] Ashraf M. A. Iqbal M. Rasheed R. Hussain I. Riaz M. Arif M. S. (2018). Environmental stress and secondary metabolites in plants: an overview. Plant metabolites Regul. under Environ. Stress Ahmad P. Ahanger M. A. Singh V. P. Tripathi D. K. Alam P. Alyemeni M. N. (Cambridge, Massachusetts, US: Academic Press), 153–167. doi: 10.1016/B978-0-12-812689-9.00008-X

[B8] Bates L. S. Waldren R. P. Teare I. (1973). Rapid determination of free proline for water-stress studies. Plant Soil 39, 205–207. doi: 10.1007/BF00018060

[B9] Bistgani Z. E. Hashemi M. Dacosta M. Craker L. Maggi F. Morshedloo M. R. (2019). Effect of salinity stress on the physiological characteristics, phenolic compounds and antioxidant activity of thymus vulgaris l. and thymus daenensis celak. Ind. Crops Prod 135, 311–320. doi: 10.1016/j.indcrop.2019.04.055

[B10] Biswas S. Koul M. Bhatnagar A. K. (2011). Effect of salt, drought and metal stress on essential oil yield and quality in plants. Nat. Prod. Commun. 6, 1934578X1100601036. doi: 10.1177/1934578X1100601036 22164806

[B11] Bouwmeester H. J. Gershenzon J. Konings M. C. Croteau R. (1998). Biosynthesis of the monoterpenes limonene and carvone in the fruit of caraway: I. demonstration of enzyme activities and their changes with development. Plant Physiol. 117, 901–912. doi: 10.1104/pp.117.3.901 9662532 PMC34944

[B12] Brady K. U. Kruckeberg A. R. Bradshaw J. H.D. (2005). Evolutionary ecology of plant adaptation to serpentine soils. Annu. Rev. Ecol. Evol. Syst. 36, 243–266. doi: 10.1146/annurev.ecolysis.35.021103.105730

[B13] Brand-Williams W. Cuvelier M.-E. Berset C. (1995). Use of a free radical method to evaluate antioxidant activity. LWT - Food Sci. Technol. 28, 25–30. doi: 10.1016/S0023-6438(95)80008-5

[B14] Cirillo C. De Micco V. Arena C. Carillo P. Pannico A. De Pascale S.. (2019). Biochemical, physiological and anatomical mechanisms of adaptation of callistemon citrinus and viburnum lucidum to NaCl and CaCl2 salinization. Front. Plant Sci. 10. doi: 10.3389/fpls.2019.00742 PMC6558163 31214238

[B15] Çoban Ö. Baydar N. G. (2016). Brassinosteroid effects on some physical and biochemical properties and secondary metabolite accumulation in peppermint (Mentha piperita l.) under salt stress. Ind. Crops Prod 86, 251–258. doi: 10.1016/j.indcrop.2016.03.049

[B16] Delfine S. Velikova V. B. Mastrodonato F. (2022). Soil-mulching influence on spearmint oil yield, ecophysiological activities and essential-oil content in rainfed environment of southern Italy. Agronomy 12, 1521. doi: 10.3390/agronomy12071521

[B17] De Micco V. Arena C. Amitrano C. Rouphael Y. De Pascale S. Cirillo C. (2021). Effects of NaCl and CaCl2 salinization on morpho-anatomical and physiological traits of potted callistemon citrinus plants. Forests 12, 1666. doi: 10.3390/f12121666

[B18] Dichala O. Giannakoula A. E. Therios I. (2022). Effect of salinity on physiological and biochemical parameters of leaves in three pomegranate (Punica granatum l.) cultivars. Appl. Sci. 12, 8675. doi: 10.3390/app12178675

[B19] Dionisio-Sese M. L. Tobita S. (1998). Antioxidant responses of rice seedlings to salinity stress. Plant Sci. 135, 1–9. doi: 10.1016/S0168-9452(98)00025-9

[B20] El-Alakmy A. A. Hassan H. Badawy M. Y. Ali M. A. (2017). Improving productivity of wild mint (mentha longifolia l.) plants by using humic acid under saline water irrigation conditions. SINJAS 6, 89–100. doi: 10.21608/SINJAS.2017.78694

[B21] El-Bassiouny H. M. Bekheta M. (2005). Effect of salt stress on relative water content, lipid peroxidation, polyamines, amino acids and ethylene of two wheat cultivars. Int. J. Agric. Biol. 7, 363–368. http://www.ijab.org

[B22] El Sabagh A. Islam M. S. Skalicky M. Ali Raza M. Singh K. Anwar Hossain M.. (2021). Salinity stress in wheat (Triticum aestivum l.) in the changing climate: adaptation and management strategies. Front. Agron. 3. doi: 10.3389/fagro.2021.6619

[B23] Fageria N. K. Stone L. F. Santos A. B. D. (2012). Breeding for salinity tolerance. Plant Breed. abiotic Stress tolerance (Berlin, Heidelberg: Springer), 103–122. doi: 10.1007/978-3-642-30553-5_7

[B24] Farsaraei S. Moghaddam M. Pirbalouti A. G. (2020). Changes in growth and essential oil composition of sweet basil in response of salinity stress and superabsorbents application. Sci. Hortic. 271, 109465. doi: 10.1016/j.scienta.2020.109465

[B25] Farzaei F. Morovati M. R. Farjadmand F. Farzaei M. H. (2017a). A mechanistic review on medicinal plants used for diabetes mellitus in traditional Persian medicine. Evid.-Based Complementary Altern. Med. 22, 944–955. doi: 10.1177/2156587216686461 PMC5871259 29228789

[B26] Farzaei M. H. Bahramsoltani R. Ghobadi A. Farzaei F. Najafi F. (2017b). Pharmacological activity of mentha longifolia and its phytoconstituents. J. Tradit. Chin. Med. Sci. 37, 710–720. doi: 10.1016/S0254-6272(17)30327-8 32188234

[B27] Geilfus C.-M. (2018). Review on the significance of chlorine for crop yield and quality. Plant Sci. 270, 114–122. doi: 10.1016/j.plantsci.2018.02.014 29576063

[B28] Gengmao Z. Quanmei S. Yu H. Shihui L. Changhai W. (2014). The physiological and biochemical responses of a medicinal plant (Salvia miltiorrhiza l.) to stress caused by various concentrations of NaCl. PloS One 9, e89624. doi: 10.1371/journal.pone.0089624 24586918 PMC3934908

[B29] George E. Rolf S. John R. (2013). Methods of soil, plant, and water analysis: A manual for the West Asia and north Africa region Vol. 244 (Beirut, Lebanon: International Center for Agricultural Research in the Dry Areas (ICARDA).

[B30] Ghassemi F. Jakeman A. J. Nix H. A. (1995). Salinisation of land and water resources: human causes, extent,management and case studies (Wallingford, UK: CAB international). Available at: https://www.cabdirect.org/cabdirect/abstract/19976767459.

[B31] Gomes M. A. D. C. Pestana I. A. Santa-Catarina C. Hauser-Davis R. A. Suzuki M. S. (2017). Salinity effects on photosynthetic pigments, proline, biomass and nitric oxide in salvinia auriculata aubl. Acta Limnol. Bras. 29. doi: 10.1590/S2179-975X4716

[B32] Guo W. Chen S. Hussain N. Cong Y. Liang Z. Chen K. (2015). Magnesium stress signaling in plant: just a beginning. Plant Signal. Behav. 10, e99228. doi: 10.4161/15592324.2014.992287 PMC4622836 25806908

[B33] Guo W. Cong Y. Hussain N. Wang Y. Liu Z. Jiang L.. (2014). The remodeling of seedling development in response to long-term magnesium toxicity and regulation by ABA–DELLA signaling in arabidopsis. Plant Cell Physiol. 55, 1713–1726. doi: 10.1093/pcp/pcu102 25074907

[B34] Hazzoumi Z. Moustakime Y. Joutei K. A. (2015). Effect of arbuscular mycorrhizal fungi (AMF) and water stress on growth, phenolic compounds, glandular hairs, and yield of essential oil in basil (Ocimum gratissimum l). Chem. Biol. Technol. 2, 1–11. doi: 10.1186/s40538-015-0035-3

[B35] Henson I. Mahalakshmi V. Bidinger F. Alagarswamy G. (1981). Genotypic variation in pearl millet (Pennisetum americanum (L.) leeke), in the ability to accumulate abscisic acid in response to water stress. J. Exp. Bot. 32, 899–910. https://www.jstor.org/stable/23690235

[B36] Hintz T. Matthews K. K. Di R. (2015). The use of plant antimicrobial compounds for food preservation. BioMed. Res. Int. 2015, 1–12. doi: 10.1155/2015/246264 PMC4619768 26539472

[B37] Hopmans J. W. Qureshi A. Kisekka I. Munns R. Grattan S. Rengasamy P.. (2021). Critical knowledge gaps and research priorities in global soil salinity. Adv. Agron. 169, 1–191. doi: 10.1016/bs.agron.2021.03.001

[B38] Houimli S. I. M. Denden M. Mouhandes B. D. (2010). Effects of 24-epibrassinolide on growth, chlorophyll, electrolyte leakage and proline by pepper plants under NaCl-stress. EurAsia J. Biosci. 4, 96–104. doi: 10.5053/ejobios.2010.4.0.12

[B39] Iqbal Z. Iqbal M. S. Hashem A. Abd Allah E. F. Ansari M. I. (2021). Plant defense responses to biotic stress and its interplay with fluctuating dark/light conditions. Front. Plant Sci. 12. doi: 10.3389/fpls.2021.63181 PMC7982811 33763093

[B40] Ivy N. Mukherjee T. Bhattacharya S. Ghosh A. Sharma P. (2022). Arsenic contamination in groundwater and food chain with mitigation options in Bengal delta with special reference to Bangladesh. Environ. Geochem Health, 1–27. doi: 10.1007/s10653-022-01330-9 35841495

[B41] Jabeen Z. Fayyaz H. A. Irshad F. Hussain N. Hassan M. N. Li J.. (2021). Sodium nitroprusside application improves morphological and physiological attributes of soybean (Glycine max l.) under salinity stress. PloS One 16, e0248207. doi: 10.1371/journal.pone.0248207 33861749 PMC8051766

[B42] Jedrzejczyk I. Rewers M. (2018). Genome size and ISSR markers for mentha L.(Lamiaceae) genetic diversity assessment and species identification. Ind. Crops Prod 120, 171–179. doi: 10.1016/j.indcrop.2018.04.062

[B43] Jezek M. Geilfus C.-M. Bayer A. Mühling K.-H. (2015). Photosynthetic capacity, nutrient status, and growth of maize (Zea mays l.) upon MgSO4 leaf-application. Front. Plant Sci. 5. doi: 10.3389/fpls.2014.00781 PMC4288286 25620973

[B44] Kalefetoğlu T. Ekmekci Y. (2005). The effects of drought on plants and tolerance mechanisms. Gazi Univ. J. Sci. 18, 723–740. https://dergipark.org.tr/en/pub/gujs/issue/7416/97089

[B45] Khare S. Singh N. Singh A. Hussain I. Niharika K. Yadav V.. (2020). Plant secondary metabolites synthesis and their regulations under biotic and abiotic constraints. J. Plant Biol. 63, 203–216. doi: 10.1007/s12374-020-09245-7

[B46] Kobayashi H. Masaoka Y. Sato S. (2005). Effects of excess magnesium on the growth and mineral content of rice and echinochloa. Plant Prod. Sci. 8, 38–43. doi: 10.1626/pps.8.38

[B47] Krzyzanowska J. Janda B. Pecio L. Stochmal A. Oleszek W. Czubacka A.. (2011). Determination of polyphenols in mentha longifolia and m. piperita field-grown and *in vitro* plant samples using UPLC-TQ-MS. J. AOAC Int. 94, 43–50. doi: 10.1093/jaoac/94.1.43 21391480

[B48] Kulak M. Gul F. Sekeroglu N. (2020). Changes in growth parameter and essential oil composition of sage (Salvia officinalis l.) leaves in response to various salt stresses. Ind. Crops Prod 145, 112078. doi: 10.1016/j.indcrop.2019.112078

[B49] Kumar B. Kumar U. Yadav H. K. (2015). Identification of EST–SSRs and molecular diversity analysis in mentha piperita. Ind. Crops Prod 3, 335–342. doi: 10.1016/j.cj.2015.02.002

[B50] Kumar S. Li G. Yang J. Huang X. Ji Q. Liu Z.. (2021). Effect of salt stress on growth, physiological parameters, and ionic concentration of water dropwort (Oenanthe javanica) cultivars. Front. Plant Sci. 12. doi: 10.3389/fpls.2021.66040 PMC8256277 34234795

[B51] Lazarević B. Šatović Z. Nimac A. Vidak M. Gunjača J. Politeo O.. (2021). Application of phenotyping methods in detection of drought and salinity stress in basil (Ocimum basilicum l.). Front. Plant Sci. 12. doi: 10.3389/fpls.2021.62944 PMC7929983 33679843

[B52] Leblebİcİ S. Sülüş Ş. Kiliçaslan G.Ç. (2021). Effect of salt stress (Potassium chloride) on the ecological and physiological characteristics of safflower (Carthamus tinctorius l.) varieties. J. anatol. Environ. Anim. Sci. 6, 441–448. doi: 10.35229/jaes.958049

[B53] Lichtenthaler H. K. (1987). [34] chlorophylls and carotenoids: pigments of photosynthetic biomembranes. Methods Enzymol 148, 350–382. doi: 10.1016/0076-6879(87)48036-1

[B54] Ma C. Bian C. Liu W. Sun Z. Xi X. Guo D.. (2022). Strigolactone alleviates the salinity-alkalinity stress of malus hupehensis seedlings. Front. Plant Sci. 13. doi: 10.3389/fpls.2022.901782 PMC9354494 35937337

[B55] Machado R. M. A. Serralheiro R. P. (2017). Soil salinity: effect on vegetable crop growth. management practices to prevent and mitigate soil salinization. Sci. Hortic. 3, 30. doi: 10.3390/horticulturae3020030

[B56] Miransari M. Mahdavi S. Smith D. (2021). The biological approaches of altering the growth and biochemical properties of medicinal plants under salinity stress. Appl. Microbiol. Biotechnol. 105, 7201–7213. doi: 10.1007/s00253-021-11552-z 34519854

[B57] Moetamedipoor S. A. Saharkhiz M. J. Khosravi A. R. Jowkar A. (2021). Essential oil chemical diversity of Iranian mints. Ind. Crops Prod 172, 114039. doi: 10.1016/j.indcrop.2021.114039

[B58] Mohammadi M. H. Z. Panahirad S. Navai A. Bahrami M. K. Kulak M. Gohari G. (2021). Cerium oxide nanoparticles (CeO2-NPs) improve growth parameters and antioxidant defense system in Moldavian balm (Dracocephalum moldavica l.) under salinity stress. Plant Stress 1, 100006. doi: 10.1016/j.stress.2021.100006

[B59] Narjary B. Jangra P. Abhishek R. Kumar N. Raju R. Thimappa K.. (2017) Quantitative assessment of soil salinity using electromagnetic induction technique and geostatistical approach. Available at: https://cssri.res.in/site/pdfs/2017/JSSWQ-Vol.9-No.2-2017.pdf#page=15.

[B60] Negacz K. Malek Ž. De Vos A. Vellinga P. (2022). Saline soils worldwide: Identifying the most promising areas for saline agriculture. J. Arid Environ. 203, 104775. doi: 10.1016/j.jaridenv.2022.104775

[B61] Okut N. Yagmur M. Selcuk N. Yildirim B. (2017). Chemical composition of essential oil of mentha longifolia l. subsp. longifolia growing wild. Pak J. Bot. 48, 525–529. https://hdl.handle.net/20.500.12513/4164

[B62] Paravar A. Maleki Farahani S. Rezazadeh A. (2022). Lallemantia iberica and lallemantia royleana: The effect of mycorrhizal fungal inoculation on growth and mycorrhizal dependency under sterile and non-sterile soils. Commun. Soil Sci. Plant Anal. 53, 880–891. doi: 10.1080/00103624.2022.2034844

[B63] Patonay K. Szalontai H. Csugány J. Szabó-Hudák O. Kónya E. P. Németh É.Z. (2019). Comparison of extraction methods for the assessment of total polyphenol content and *in vitro* antioxidant capacity of horsemint (Mentha longifolia (L.) l.). J. Appl. Res. Med. Aromat. Plants 15, 100220. doi: 10.1016/j.jarmap.2019.100220

[B64] Qadir M. Quillérou E. Nangia V. Murtaza G. Singh M. Thomas R. J.. (2014). Economics of salt-induced land degradation and restoration. Natural Resour. Forum 38, 282–295. doi: 10.1111/1477-8947.12054

[B65] Rasheed D. Azorji J. Wisal A. S. Nawchukwu M. Nwachukwu C. (2020). Effect of potassium chloride (KCl) on biochemical and morphological parameters of triticum aestivum l. Asian J. Res. Bot. 3, 10–17. doi: 10.13140/RG.2.2.24139.41769

[B66] Razavi S. Zarrini G. Molavi G. (2012). The evaluation of some biological activity of mentha longifolia (L.) huds growing wild in Iran. Pharmacologia 3, 535–538. doi: 10.17311/pharmacologia.2012.535.538

[B67] Segev D. Nitzan N. Chaimovitsh D. Eshel A. Dudai N. (2012). Chemical and morphological diversity in wild populations of mentha longifolia in Israel. Chem. Biodivers. 9, 577–588. doi: 10.1002/cbdv.201100108 22422525

[B68] Selvaraj V. Sankar J. (2010). Characterisation of magnesium toxicity, its influence on amino acid synthesis pathway and biochemical parameters of tea. Res. J. Phytochem. 4, 67–77. doi: 10.3923/rjphyto.2010.67.77

[B69] Shahid S. A. Zaman M. Heng L. (2018). “Soil salinity: Historical perspectives and a world overview of the problem,” in Guideline for salinity assessment, mitigation and adaptation using nuclear and related techniques (Cham: Springer). doi: 10.1007/978-3-319-96190-3_2

[B70] Shrivastava P. Kumar R. (2015). Soil salinity: A serious environmental issue and plant growth promoting bacteria as one of the tools for its alleviation. Saudi J. Biol. Sci. 22, 123–131. doi: 10.1016/j.sjbs.2014.12.001 25737642 PMC4336437

[B71] Singh R. Luxmi S. Charak A. Gochar R. Kumar A. Gandhi S. G.. (2022). Effects of intermittent drought on the essential oil yield, contents, and nutrient status of mentha longifolia (L.) huds. J. Essent. Oil-Bear Plants 25, 626–638. doi: 10.1080/0972060X.2022.2091957

[B72] Soilhi Z. Rhimi A. Heuskin S. Fauconnier M.-L. Mekki M. (2019). Essential oil chemical diversity of Tunisian mentha spp. collection. Ind. Crops Prod 131, 330–340. doi: 10.1016/j.indcrop.2019.01.041

[B73] Szymańska S. Lis M. I. Piernik A. Hrynkiewicz K. (2022). Pseudomonas stutzeri and kushneria marisflavi alleviate salinity stress-associated damages in barley, lettuce, and sunflower. Front. Microbiol. 13. doi: 10.3389/fmicb.2022.788 PMC8957930 35350624

[B74] Taarit M. B. Msaada K. Hosni K. Hammami M. Kchouk M. E. Marzouk B. (2009). Plant growth, essential oil yield and composition of sage (Salvia officinalis l.) fruits cultivated under salt stress conditions. Ind. Crops Prod 30, 333–337. doi: 10.1016/j.indcrop.2009.06.001

[B75] Tang N. Li Y. Chen L. S. (2012). Magnesium deficiency–induced impairment of photosynthesis in leaves of fruiting citrus reticulata trees accompanied by up-regulation of antioxidant metabolism to avoid photo-oxidative damage. J. Plant Nutr. Soil Sci. 175, 784–793. doi: 10.1002/jpln.201100329

[B76] Thorne S. J. Hartley S. E. Maathuis F. J. (2020). Is silicon a panacea for alleviating drought and salt stress in crops? Front. Plant Sci. 11. doi: 10.3389/fpls.2020.01221 PMC7461962 32973824

[B77] Vafadar F. Amooaghaie R. Ehsanzadeh P. Ghanadian M. (2020). Salinity stress alters ion homeostasis, antioxidant activities and the production of rosmarinic acid, luteolin and apigenin in dracocephalum kotschyi boiss. Biologia 75, 2147–2158. doi: 10.2478/s11756-020-00562-3 32593901

[B78] Valko M. Rhodes C. Moncol J. Izakovic M. Mazur M. (2006). Free radicals, metals and antioxidants in oxidative stress-induced cancer. Chem.-Biol. Interact. 160, 1–40. doi: 10.1016/j.cbi.2005.12.009 16430879

[B79] Voko M. P. Kulkarni M. G. Ngoroyemoto N. Gupta S. Finnie J. F. Van Staden J. (2022). Vermicompost leachate, seaweed extract and smoke-water alleviate drought stress in cowpea by influencing phytochemicals, compatible solutes and photosynthetic pigments. Plant Growth Regul. 97, 327–342. doi: 10.1007/s10725-022-00815-y

[B80] White P. J. Broadley M. R. (2003). Calcium in plants. Ann. Bot. 92, 487–511. doi: 10.1093/aob/mcg164 12933363 PMC4243668

[B81] Wyka T. P. Oleksyn J. Żytkowiak R. Karolewski P. Jagodziński A. Reich P. B. (2012). Responses of leaf structure and photosynthetic properties to intra-canopy light gradients: a common garden test with four broadleaf deciduous angiosperm and seven evergreen conifer tree species. Oecologia 170, 11–24. doi: 10.1007/s00442-012-2279-y 22349756 PMC3422461

[B82] Xiao F. Xu T. Lu B. Liu R. (2020). Guidelines for antioxidant assays for food components. Food Front. 1, 60–69. doi: 10.1002/fft2.10

[B83] Yang Y. Guo Y. (2018). Unraveling salt stress signaling in plants. J. Integr. Plant Biol. 60, 796–804. doi: 10.1111/jipb.12689 29905393

[B84] Yao S. Chen S. Xu D. Lan H. (2010). Plant growth and responses of antioxidants of chenopodium album to long-term NaCl and KCl stress. Plant Growth Regul. 60, 115–125. doi: 10.1007/s10725-009-9426-4

[B85] Zhang Q.-H. Sairebieli K. Zhao M.-M. Sun X.-H. Wang W. Yu X.-N.. (2020). Nutrients have a different impact on the salt tolerance of two coexisting suaeda species in the yellow river delta. Wetlands 40, 2811–2823. doi: 10.1007/s13157-020-01382-6

